# Mechanical Strain Induces Transcriptomic Reprogramming of Saphenous Vein Progenitors

**DOI:** 10.3389/fcvm.2022.884031

**Published:** 2022-05-27

**Authors:** Davide Maselli, Gloria Garoffolo, Giada Andrea Cassanmagnago, Rosa Vono, Matthijs S. Ruiter, Anita C. Thomas, Paolo Madeddu, Maurizio Pesce, Gaia Spinetti

**Affiliations:** ^1^IRCCS MultiMedica, Milan, Italy; ^2^Translational Health Sciences, Bristol Medical School, University of Bristol, Bristol, United Kingdom; ^3^Unità di Ingegneria Tissutale Cardiovascolare, Centro Cardiologico Monzino IRCCS, Milan, Italy; ^4^IRCCS Humanitas Research Hospital, Rozzano, Italy; ^5^Department of Biomedical Sciences, Humanitas University, Pieve Emanuele, Italy

**Keywords:** saphenous vein progenitors, intimal hyperplasia, bypass graft, mechanosensitivity, AMIGO2

## Abstract

Intimal hyperplasia is the leading cause of graft failure in aortocoronary bypass grafts performed using human saphenous vein (SV). The long-term consequences of the altered pulsatile stress on the cells that populate the vein wall remains elusive, particularly the effects on saphenous vein progenitors (SVPs), cells resident in the vein adventitia with a relatively wide differentiation capacity. In the present study, we performed global transcriptomic profiling of SVPs undergoing uniaxial cyclic strain *in vitro*. This type of mechanical stimulation is indeed involved in the pathology of the SV. Results showed a consistent stretch-dependent gene regulation in cyclically strained SVPs vs. controls, especially at 72 h. We also observed a robust mechanically related overexpression of Adhesion Molecule with Ig Like Domain 2 (AMIGO2), a cell surface type I transmembrane protein involved in cell adhesion. The overexpression of AMIGO2 in stretched SVPs was associated with the activation of the transforming growth factor β pathway and modulation of intercellular signaling, cell-cell, and cell-matrix interactions. Moreover, the increased number of cells expressing AMIGO2 detected in porcine SV adventitia using an *in vivo* arterialization model confirms the upregulation of AMIGO2 protein by the arterial-like environment. These results show that mechanical stress promotes SVPs' molecular phenotypic switching and increases their responsiveness to extracellular environment alterations, thus prompting the targeting of new molecular effectors to improve the outcome of bypass graft procedure.

## Introduction

Bypass grafting surgery is the main treatment for coronary artery disease, which represents the leading cause of morbidity and mortality in the industrialized western world (1, 2). Every year about one million surgical revascularization procedures are performed worldwide, and the saphenous vein (SV) remains the most widely used conduit as bypass graft (3). The reasons for this preference are the ease and rapid SV harvesting technique and its relatively higher length compared to radial or mammary arteries, which ensures enough supply for “multi-vessel” pathology (4). However, up to 20–50% of vein grafts will require intervention within 5 years due to the development of graft stenosis caused by intimal hyperplasia (IH) (5, 6). A better understanding of the development and progression of vein graft disease is crucial for the development of new treatments. The causes of the progressive thickening of the tunica intima of SVs, used as a coronary bypass graft, leading to IH can be partially identified in surgical mismanagement at the time of harvesting, for which recently has been introduced “no-touch” harvesting technique (7). A better outcome in terms of SV graft patency has been also observed when coronary artery bypass graft surgery is performed in off-pump mode (8). However, IH represents a long-term consequence of vein adaptation to coronary blood flow (8) consisting of a change from a constant pressure (5–10 mmHg) and a steady flow, to a counter pulsed 120/80 mmHg and pulsatile flow, with a circumferential strain of 10–15% (9, 10). Under these conditions, wall shear stress on the endothelial monolayer increases by four times, and the quasi-steady venous flow rises to a mean flow rate of 250 ml/min (10). Opposite to arteries, the SV wall is incompliant at high pressures resulting in a blood flow rate in SV bypass graft 5–10 times higher than in arterial bypass graft (11–13). As a consequence, the cells in the tunica media of the vessel are exposed to severe stretching causing an increase in proliferation, changes in the extracellular matrix, and phenotype switching (14–16). There is an increasing number of evidence about the detrimental effect of mechanical stress on the tunica adventitia and strain-related activation of cells dwelling in the proximity of the *vasa vasorum* of the SV' bypass graft (17–20). In particular, it has been observed that paracrine signals, established in an arterial-like pressure setting, might activate a multipotent population of cells, known as saphenous vein progenitors (SVPs), which uphold differentiation capacity in aged cardiovascular patients (21). Of note, we recently showed that mechanical straining of SV-derived smooth muscle cells (SMCs) determines their transition from a contractile to a secretory phenotype with associated release of the matricellular protein Thrombospondin-1 (TSP-1), which consequently induces the migration and proliferation of cells with SVP phenotype (19). Given the importance of tissue mechanics for the progression of vein graft disease and the potential role of SVP in SV remodeling, in this study, we aimed to assess the strain-related modification of SVPs. Using an RNAseq-based approach we evaluated whether mechanical strain could induce a specific phenotypic modification of SVPs. To investigate stress-related phenotypic changes we exposed primarily isolated SVPs to *in vitro* uniaxial mechanical strain. Genome-wide transcriptional alterations were examined using RNA-seq and analyzed for specific function/gene regulations.

## Materials and Methods

### Ethics

The experimental investigation on human-derived SVPs was approved by the local Ethical Committee at Centro Cardiologico Monzino, IRCCS. Twelve patients recruited for the study were, females or males undergoing surgical removal of saphenous vein because of varicosity. All tissues used in the study are surgical leftovers. Exclusion criteria include concomitant neoplastic, infectious, connective tissue or inflammatory diseases, pregnancy. The Research Ethics Committee approved the study which was performed according to the ethical principles recorded in the 1964 Declaration of Helsinki and later amendments. All the subjects gave written informed consent to participate. The main clinical characteristics of the participants are included in Supplementary Table 1.

### Isolation of SVPs

Isolation of cells for *in vitro* experiments was performed as previously described, using two consecutive immunomagnetic selections to obtain CD31^negative^/CD34^positive^ homogenous cell populations from saphenous vein, known as SVPs (21). After saphenectomy, veins were washed in PBS containing Penicillin/Streptomycin 100 U/mL (PBS + P/S). Saphenous vein walls were finely shredded using scissors and digested with Liberase Blendzyme 2 (Roche) diluted 2 mg/ml in Dulbecco Modified Eagle's Medium (DMEM, GIBCO) for 4h at 37°C. The minced tissue was serially filtered through 70 μm, 40 μm, and 30 μm cell strainers using PBS + P/S and then centrifuged at 300xg for 10 min at room temperature. SVPs were isolated as a result of two consecutive magnetic beads–assisted cell sorting (MACS, MiltenyiBiotec) using a MACS MS column, according to manufacturer's instructions. First selection was performed using CD31 magnetic beads (MiltenyiBiotec), in which the negative fraction (CD31^negative^) was retained and subjected to a second magnetic sorting performed with CD34 magnetic beads (MiltenyiBiotec). After that, CD31^negative^/CD34^positive^ cell fraction was centrifuged and resuspended in endothelial growth medium EGM-2 (Lonza). SVPs were plated on fibronectin/gelatin-coated plate (0.1% fibronectin - 0.4% gelatin in PBS) 3 × 10^3^ cells/cm^2^ and cultured at 37°C in a cell culture incubator with 5% CO_2_. Culture medium was replaced twice a week and once cells reached 80% confluency, they were trypsinized and split 1:3. Cells used in RNA-seq were between passage 3 and 5.

### Immunocytochemical Characterization

To validate the identity of isolated SVPs we performed immunocytochemical characterization of the cultured cells for expression of neural/glial antigen 2 (NG2), platelet derived growth factor receptor-β (PDGFRβ), GATA Binding Protein 4 (GATA4), CD31, CD146 and α-smooth muscle actin (α-SMA) (Supplementary Figure 1). SVPs were seeded at 5x10^3^ cells/cm^2^ on fibronectin/gelatin-coated plate chamber slides, after 24 hours (h) cells were washed with PBS and fixed with 4% PFA in PBS for 15 min at room temperature. When required (GATA4 and α-SMA), the cells were permeabilized with 0.1% Triton X-100 (Sigma-Aldrich) in PBS for 10 min at room temperature. Non-specific staining was blocked with 5% Fetal Bovine Serum (FBS) (GIBCO) in PBS for 30 min at room temperature. Following elimination of excess serum, the cells were exposed to the unconjugated primary antibodies at 4°C for 16 h: NG2 (1:100, Millipore AB-5320), PDGFRβ (1:50, Santa Cruz SC-339), GATA4 (1:100, Abcam ab61767), CD31 (1:100, R&D BBA7), CD146 (1:100, Abcam ab75769), α-SMA (1:200, Dako M0851). After washing in PBS, the appropriate fluorescent secondary antibody (Alexa Fluor) diluted 1:200 in PBS was added to the cells for 60 min at 37°C. Nuclei were stained with DAPI (Thermo Fisher Scientific)1 μg/ml for 10 min at room temperature. Photos of random fields were taken at 20x magnification with Zeiss Observer Z1 inverted microscope.

### *In vitro* Mechanical Stimulation of SVPs

To investigate the effect of mechanical strain on cultured cells, SVPs were subjected to cyclic strain using the FlexCell Tension Plus FX-5000T system. Before cell seeding, six-well uniaxial Bioflex plates were surface-coated with human fibronectin (10μg/ml) in PBS after covalent crosslinking with a crosslinking reagent (sulfosuccinimidyl 6-(4'-azido-2'-nitrophenylamino) hexanoate; Sulfo-SANPAH) at 0.2 mg/ml in Hepes 50 mM (pH 8.5), photo-activated by exposure to UV-light (365 nm). Cells were subjected to uniaxial cyclic deformation protocol (0–10% deformation, 1 Hz frequency), for 24 and 72 h [according to a protocol established in (19)], while static controls were provided by seeding an equal amount of cells, under the same atmospheric conditions, but without mechanical stimulation (Supplementary Figures 2A,B). For imaging of mechanically stimulated SVPs, cells were fixed after 72 h of uniaxial cyclic straining with 4% PFA in PBS, and thereafter stained with Phalloidin-TRITC (1:500, Sigma) and DAPI 1μg/ml (Thermo Fisher Scientific) at room temperature for 1 h.

### Total RNA Isolation

For RNA-Seq analysis, total RNA was extracted from 5 different donors using RNeasy Mini kit (Qiagen). Cultured SVPs, approximately 4×10^5^ to 5×10^5^ cells per well, were resuspended in 700 μl of QIAzol Lysis Reagent (Qiagen) and residual DNA was removed by on-column DNase digestion. Total RNA was purified following the manufacturer's instructions and quantified by using NanoDrop-1000 spectrophotometer before integrity assessment with Agilent 2100 Bioanalyzer (RNA Integrity Number values >8).

### RNA-Seq on *in vitro* Mechanical Stimulated SVPs

Next-generation sequencing experiments, including samples quality control and bioinformatics analysis, were performed by Genomix4life S.R.L. (Baronissi, Salerno, Italy). Indexed libraries were prepared from 500 ng/ea purified RNA with TruSeqStranded total RNA Sample Prep Kit (Illumina) according to the manufacturer's instructions. Libraries were quantified using the Agilent 2100 Bioanalyzer (Agilent Technologies) and Qubit fluorometer (Invitrogen Co.), then pooled such that each index-tagged sample was present in equimolar amounts, with a final concentration of the pooled samples of 2 nM. The pooled samples were subject to cluster generation and sequencing using an Illumina HiSeq 2,500 System (Illumina) in a 2 × 100 paired-end format at a final concentration of 8 pmol. The raw sequence files generated (.fastq files) underwent quality control analysis using FastQC (http://www.bioinformatics.babraham.ac.uk/projects/fastqc/) and the quality checked reads were trimmed with cutadapt v.1.10 and then aligned to the human genome (hg38 assembly) using STAR v.2.5.2, with standard parameters. Differentially expressed mRNAs were identified using DESeq2 v.1.12. Gene annotation was obtained for all known genes in the human genome, as provided by GenCode (GRCh38.p7 release 25). Using the reads mapped to the genome, we calculated the number of reads mapping to each transcript with HT Seq-count v.0.6.1. These raw read counts were then used as input to DESeq2 for calculation of normalized signal for each transcript in the samples, and differential expression was reported as Fold Change along with associated adjusted *p*-values (computed according to Benjamini-Hochberg). Raw data of RNA-Seqthat support the findings of this publication have been deposited in NCBI's Gene Expression Omnibus (22) and are accessible through GEO Series accession number GSE192712 (https://www.ncbi.nlm.nih.gov/geo/query/acc.cgi?acc=GSE192712).

### RNA-Seq Data Pre-processing and Analysis

Upon inspection of quality control features with FastQC (version 0.11.8) [Andrews (2010). FastQC: a quality control tool for high throughput sequence data. Available online at: http://www.bioinformatics.babraham.ac.uk/projects/fastqc] and MultiQC (version 1.8), the raw sequencing reads were trimmed to remove adaptor contaminations, using Cutadapt (version 1.18) with the following parameters: cutadapt-a AGATCGGAAGAGCACACGTCTGAACTCCAGTCA -A AGATCGGAAGAGCGTCGTGTAGGGAAAGAGTGT –trim-n –pair-filter=any –minimum-length 20. Genome contamination screening was performed through FastQ Screen (version v0.14.0). The sequencing reads were, then, aligned to GENCODE's human reference genome (GRCh38 primary assembly v31) and quantified at the gene level using STAR (version2.7.3a), with default parameters and allowing up to 3 mismatches.

Evaluation of sequencing alignment data was performed through Qualimap application (v.2.2.2-dev) (23), using the following analysis types: Multi-sample BAM QC, RNA-seq QC, and Counts QC.

RNA-Seq analysis was performed using DESeq2 package (version 1.22.2) (24) in the R software (version 3.6.3) [R Core Team (2020). R: A language and environment for statistical computing. R Foundation for Statistical Computing, Vienna, Austria. URL https://www.R-project.org/]. Samples were normalized for sequencing depth and RNA composition according to DESeq2 median of ratios method. Differential gene expression analysis was performed on genes that passed genefilter/DESeq2 independent filtering procedure (alpha = 0.05). Genes showing a BH-adjusted *p*-value ≤ 0.05 and log2FoldChange > 1.5 or log2FoldChange < −1.5 were considered differentially expressed (DEGs).

For visualization purposes, counts were transformed using DESeq2 regularized log-transformation. DESeq2 plotPCA function was used for PCA analysis. Samples correlations were computed using the cor.dat function from the stats package (version 3.6.3). Heatmaps were plotted using the pheatmap package (version 1.0.12) [RaivoKolde (2019). pheatmap: Pretty Heatmaps. R package version 1.0.12. https://CRAN.R-project.org/package=pheatmap] and volcano plots with the EnhancedVolcano package (version 1.4.0) [Kevin Blighe, Sharmila Rana and Myles Lewis (2019). EnhancedVolcano: Publication-ready volcano plots with enhanced coloring and labeling. R package version 1.4.0. https://github.com/kevinblighe/EnhancedVolcano] (BH-adjusted *p*-value cutoff = 0.05, FC cutoff = 1.5).

Motif-based TF prediction was performed in Cytoscape (version 3.8.0) running iRegulon App (25) on the complete set of differentially expressed genes in the dynamic vs. static conditions, with the following parameters: Motif collection = 10 K (9,713 PWMs), Track collection = 1,120 ChIP-seq tracks (ENCODE raw signals), min NEScore = 2, ROC threshold for AUC calculation (% = 3), max FDR = 0.05, Motif rankings database = 20 kb centered around TSS (7 species), Track rankings database = 20 kb centered around TSS (ChIP-seq derived).

DEGs from each contrast and the corresponding logFCs and adjusted *p-*values were uploaded to Ingenuity Pathways Analysis (IPA) software (version 60467501) (QIAGEN Inc.) (26). Core Analysis was performed using default settings: only protein-protein interaction networks, upstream regulators networks and Diseases and Biological Functions enriched pathways involving AMIGO2 were considered, along with evaluation of the IPA TGF-β signaling canonical pathway. Bubble plots were produced using ggplot2 package (version 3.3.2) [H. Wickham. ggplot2: Elegant Graphics for Data Analysis. Springer-Verlag New York, 2016.] in R.

Pathway enrichment analysis on DEGs at 24 h and 72 h was performed using Metascape (27) (Express Analysis), only pathways involving AMIGO2 gene were considered.

### Porcine *in vivo* Model of Vein Graft Remodeling

*In vivo* studies were performed with Large White-Landrace cross pigs (weight 25 to 30 kg) using the saphenous vein-to-carotid artery interposition grafting model previously described (28). This investigation was performed in accordance with the Home Office Guidance on the operation of the Animals (Scientific Procedures) Act 1986 (HMSO, London, UK; PPL numbers 30/2585 and 30/3064) and was compliant with the EU Directive 2010/63/EU and principles stated in the Guide for the Care and Use of Laboratory Animals (Institute of Laboratory Animal Resources, 1996).

The animals were subjected to unilateral or bilateral autologous saphenous vein (SV) into common carotid artery bypass grafting. In brief, pigs were anesthetized with ketamine (Ketaset, 100 mg/mL), intubated, and maintained on 1–3% halothane under spontaneous ventilation. The animal was heparinized by intravenous administration of 100 IU/kg of heparin. The long saphenous vein was harvested from the hind leg using the “no touch” technique (29), rinsed in a saline solution containing 2 IU/ml heparin and 50 μg/ml glyceryl trinitrate, and stored in the same solution at room temperature until needed. A 3-cm length of the vein was placed as an interposition graft to the internal carotid artery using continuous 7/0 Surgipro sutures. The flow was re-established and checked using a hand-held Doppler flow meter (Multidoplex II (model MD2), Huntleigh Diagnostics Ltd, Cardiff, UK), and the animals were given antibiotic (ampicillin) and analgesic (buprenorphine) before and during recovery. The pigs were anesthetized and the SV grafts were collected at the established time points (1, 3, 7, 14, and 90 days), followed by euthanasia using an intra-cardiac overdose of pentobarbital. The grafts were fixed in 4% paraformaldehyde for 16 h and then embedded in paraffin for immunohistochemical analyses. Paraffin-embedded tissue sections were dehydrated and after blocking with 6% BSA for 1 h, incubated for 16 h at 4°C with the primary antibodies: AMIGO2 (1:100, Abcam ab84416) and α-SMA (1:150, Dako M0851) to enable the identification between the medial and adventitial layer. Subsequently, sections were incubated with appropriate secondary antibodies diluted 1:200 for1 h at room temperature. Nuclei were stained with DAPI 1μg/ml for 10 min at room temperature. Digital images were obtained using an ApoTome fluorescence microscope or LSM-710 confocal scanning microscope (both Carl Zeiss, Germany). Cells positive for AMIGO2, counted in 3 fields *per* section, were expressed as a percentage of cells in the whole adventitia. Measurements and quantifications were performed using ImageJ (version 1.46r, National Institutes of Health, USA). Differences between time points were analyzed in GraphPad Prism 5 using ANOVA with Newman-Keuls *post hoc* test, with a significance level of 0.05.

### *Ex vivo* Mechanical Stimulation of Human SV

SV segments for *ex vivo* culture were supplied from the Department of Cardiovascular Surgery at Centro Cardiologico Monzino. The veins were obtained from patients undergoing coronary artery bypass operations under protocols approved by the Ethical Committees of the Centro Cardiologico Monzino (Italy). Mechanical stimulation of SVs was performed using a custom-made bioreactor tailored to reproduce the coronary mechanics. SV were harvested with a “no-touch” technique (7) and stored at 4°C in DMEM supplemented with 10% FBS, 1% L-Glutamine, and 1% P/S. The *ex vivo* culture system exploited for veins stimulation was designed by Dipartimento di Elettronica, Informazione e Bioingegneria, Politecnico di Milano and Unità di Ingegneria Tissutale of Centro Cardiologico Monzino-IRCCS in Milan (30). This culture system allowed mimicking the arterial-like stimulation with a circumferential strain applied to the SV wall typical of the coronary circulation (31). Briefly, the arterial-like flow is accomplished in 4 independent phases: i) a loading step, ii) a pulsatile stimulation step, iii) an unloading step, and iv) a recirculation step. In the first phase, the vessel is filled with DMEM, 10% FBS, 1% L-Glutamine, and 1% P/S. During the pulsatile step, the medium is put under oscillating pressure between 80 mmHg and 120 mmHg and in the third phase the medium flows out of the vessels. The fourth phase is necessary to replace the medium inside the vein, thus maintaining stable nutrients and oxygen supply (17). The culture system was placed in a standard incubator at 37°C in a 5% CO 2 atmosphere and for a culture period of 14 days. Then, SV segments were un-mounted from the culture system, fixed in formalin 37% for 16 h, and then embedded in paraffin for immunohistochemical analyses. In brief, tissue sections were subjected to heat-induced antigen retrieval with 10 mM Sodium Citrate buffer pH 6.0 at 94°C for 30 min. Non-specific binding was blocked with 20% goat serum (Sigma-Aldrich) for 30 min at room temperature. Following the elimination of excess serum, the sections were exposed to the unconjugated primary antibodies at 4°C for 16 h:CD34 (1:50, R&D AF7227), AMIGO2 (1:200, Abcam ab84416), CD31 (1:50, R&D BBA7). After washing in PBS, the appropriate fluorescent secondary antibody (Alexa Fluor) diluted 1:200 in PBS was added to the cells for 60 min at 37°C. Nuclei were stained with DAPI (Thermo Fisher Scientific)1 μg/ml for 10 min at room temperature. Photos were taken at 20x magnification with Zeiss Observer Z1 inverted microscope and reconstructed using Photoshop (Adobe) to obtain the adventitia-lumen image.

## Results

### Cyclic Uniaxial Strain Induces Changes in the Transcriptome of Human SVPs

**Figure 1:**
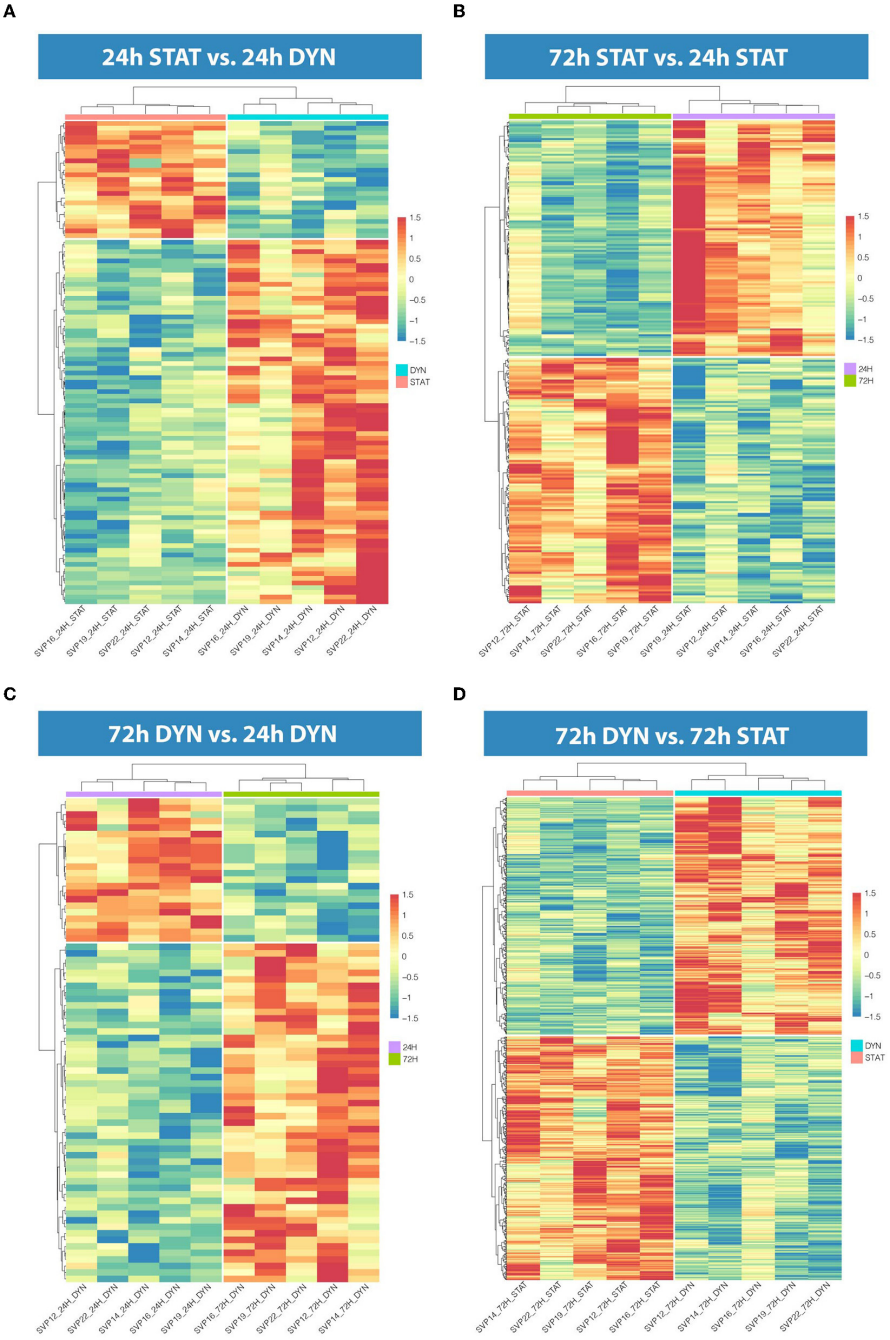
Hierarchical clustering in the four comparisons. **(A)** Heatmap representation showing 103 DEGs in 24 h dyn vs. 24 h stat. **(B)** Heatmap representation showing 245 DEGs in 72 h stat vs. 24 h stat. **(C)** Heatmap representation showing 72 DEGs in 72 h dyn vs. 24 h dyn. **(D)** Heatmap representation showing 819 DEGs in 72 h dyn vs. 72 h stat. For all heatmaps BH-adjusted *p*-value ≤ 0.05.

**Figure 2:**
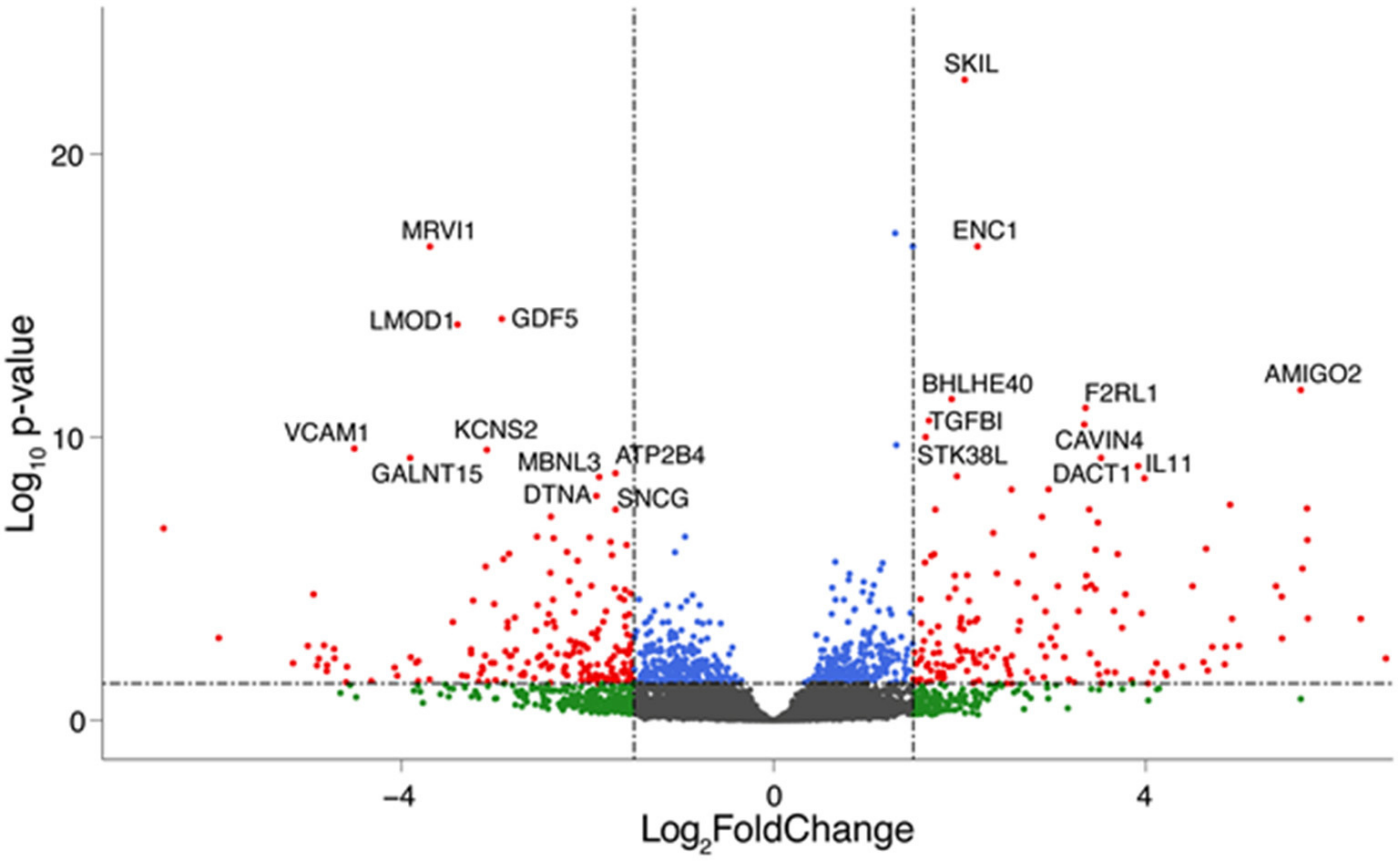
Top regulated genes after 72 h of mechanical strain. Volcano plot showing significant genes in the 72 h dyn vs. 72 h stat comparison. The top 10 upregulated and down regulated DEGs are highlighted. Genes are colored if they pass the thresholds for BH-adjusted *p*-value and/or LogFC (blue if BH-adjusted *p*-value ≤ 0.05, green if logFC < −1.5 or logFC> 1.5, red if they pass both thresholds).

To assess the effect of mechanical stress on SVP transcriptome, we subjected cells to cyclic uniaxial strain (defined as dynamic, dyn, from now on) for 24 and 72 h compared to standard culture (static, stat) and performed RNA-Seq. This *in vitro* system allowed us to identify the early alterations in the transcription, underscoring the mechanisms associated. First, we conducted an unsupervised clustering of the results based on the top 100 most variables expressed genes in the different culture conditions. This analysis and principal component analysis (PCA) showed no stretch-related groupings among replicates (Supplementary Figures 3A,B). However, since the sequencing depth allowed us to quantify the expression of 28,624 genes that represent <50% of the ones annotated for *Homo sapiens* and include genes expressed at low levels, results may be non-consistent when performing differential expression analysis. Therefore, we next conducted a selection of genes expressed at reliable levels according to DESeq2 independent filtering procedure. The four datasets resulting from this filtering were paired in four comparisons to perform the differential expression analysis as follows: 1) 24 h dyn vs. 24 h stat, and 2) 72 h dyn vs. 72 h stat assessed the effect of strain at the two different time points 24 and 72 h; 3) 72 h stat vs. 24 h stat, and 4) 72 h dyn vs. 24 h dyn evaluated the effect of time on the culture conditions. These comparisons led to the following number of differentially expressed genes (DEGs): 1) 103 DEGs (24 hdyn vs. 24 h stat), 2) 819 DEGs (72 h dyn vs. 72 h stat), 3) 245 DEGs (72 h stat vs. 24 h stat), and 4) 72 DEGs in (72 h dyn vs. 24 h dyn) (Supplementary Table 2). We used hierarchical cluster analysis to assess the relationships between the DEGs in the four experimental conditions (Figure 1). As shown, the heatmaps built using the four different datasets indicated a major prevalence of DEGs in the static vs. dynamic conditions at 72 h. In addition, coherent differential regulation of genes was already observed at an earlier time point in dynamically strained vs. control cells for 24 h (Figure 1A). Moreover, a time-dependent effect both in static and dynamic conditions was observed (Figures 1B,C). The higher number of DEGs resulting from the comparison between dynamic *vs*. static conditions at 72 h prompted us to further analyze the identity of the top-score genes up/downmodulated in this specific condition and to explore whether these genes were represented already at the earlier time point (24 h of stimulation). To this aim, we listed the 10 most up-regulated and the 10 most down-regulated genes in the 72 h dyn vs. 72 h stat condition (Table 1). These genes can be easily visualized in a volcano plot displaying the 819 DEGs arranged by fold change and Benjamini-Hochberg (BH)-adjusted *p*-value (Figure 2), they all had significantly higher expression (at least 1.5-fold more) than static controls along with a significant *p*-value (BH-adjusted *p*-value ≤ 0.05). In addition, Table 2 shows the top regulated DEGs in the 24 h dyn vs. 24 h stat conditions. Only five genes were present in the top regulated lists at both time points representing candidates consistently regulated by mechanical stress in SVPs: Adhesion Molecule with Ig Like Domain 2 (*AMIGO2*), Serine/threonine-protein kinase 38-like (*STK38L)*, Caveolae Associated Protein 4 (*CAVIN4)*, Growth differentiation factor 5 (*GDF5)* and Polypeptide N-acetylgalactosaminyltransferase 15 (*GALNT15)* (Table 3).

**Table 1:** Top scored DEGs 72 h dyn vs. 72 h stat.

**Top10 up-regulated DEGs – 72 h ON vs. 72 h OFF**			**Top10 down-regulated DEGs – 72 h ON vs. 72 h OFF**		
**Gene**	**Log_**2**_FoldChange**	***P*-value BH-adjusted**	**Gene**	**Log_**2**_FoldChange**	***P*-value BH-adjusted**
SKIL	2.05	2.35E-23	MRVI1	−3.69	1.84E-17
ENC1	2.19	1.82E-17	GDF5	−2.92	6.60E-15
AMIGO2	5.66	2.14E-12	LOMD1	−3.39	1.04E-14
BHLHE40	1.91	4.50E-12	VCAM1	−4.51	2.52E-10
F2RL1	3.35	9.19E-12	KCNS2	−3.08	2.79E-10
TGFB1	1.66	2.58E-11	GALNT15	3.91	5.39E-10
CAVIN4	3.33	3.55E-11	ATP2B4	−1.70	1.87E-09
STK38L	1.63	9.84E-11	MBNL3	−1.87	2.53E-09
DACT1	3.51	5.39E-10	DTNA	−1.90	1.18E-08
IL11	3.91	1.04E-09	SNCG	−1.70	3.58E-08

**Table 2:** Top scored DEGs 24 h dyn vs. 24 h stat.

**Top10 up-regulated DEGs – 24 h ON vs. 24 h OFF**			**Top10 down-regulated DEGs – 24 h ON vs. 24 h OFF**		
**Gene**	**Log_**2**_FoldChange**	***P*-value BH-adjusted**	**Gene**	**Log_**2**_FoldChange**	***P*-value BH-adjusted**
CAVIN4	2.91	1.23E-04	ACKR4	−2.32	1.23E-04
AMIGO2	4.49	1.23E-04	GALNT15	−2.55	5.76E-04
EDN1	2.91	1.58E-04	ANKRD33B	−2.48	8.57E-03
KIA A 1755	2.68	4.60E-04	PDE7B	−1.55	1.46E-02
MIR503HG	2.73	5.75E-04	IFIT1	−1.67	1.46E-02
SMAD7	1.58	7.37E-04	FAM107A	−3.44	1.92E-02
TSPAN2	3.17	8.21E-04	TOX	−1.76	2.89E-02
COL7A1	2.42	1.23E-03	CENPP	−1.52	3.12E-02
STK38L	1.73	1.71E-03	ZNF367	−1.74	3.80E-02
ANGPTL4	1.62	2.78E-03	GDF5	−1.54	4.41E-02

**Table 3:** DEGs consistently regulated by mechanical stress.

**Gene**	**Gene ID**	**Function**	**Log**_**2**_ **FoldChange**	
			**24 h**	**72 h**
AMIGO2	347902	This gene encodes a cell receptor involved in axon extension and migration. Also described as a pro-survival factor in endothelial cells subjected to hypoxia and regulator of tumor cell adhesion and formation of metastases.	**4.49**	**5.66**
STK38L	23012	The encoded protein is a serine/threonine kinase 38 like, inmplicated in neuronal cytoskeletal development, neurite outgrowth and synaptic remodeling.	**1.73**	**1.63**
CAVIN4	347273	Cavin-4 protein modulates the morphology of formed caveolae, results activated the extracellular signal-regulated kinase pathway, influencing skeletal muscle differentiation, and to activate RhoA pathway, modulating cardiac function.	**2.91**	**3.33**
GDF5	8200	This is one of the earliest genes expressed in the embryonic joint interzone, fated to give rise to joint tissues. Gdf5-lineage mesenchymal stromal/stem cells are involved in cartilage repair.	**−1.54**	**−2.92**
GALNT15	117248	The encoded protein catalyzes the initial reaction in O-linked oligosaccharide biosynthesis	**−2.55**	**−3.9**

### Characterization of AMIGO2 Regulation Induced by Mechanical Stress in SVPs

Among the genes that appear to be involved in vascular biology/pathology, our interest focused on *AMIGO2*, a gene encoding for a cell surface type I transmembrane receptor, which is a member of a novel class of leucine-rich repeat (LRR) and Ig superfamily proteins (32). AMIGO2 was identified for the first time in 2003, as an adhesion molecule necessary for the development of the axonal tract of neurons (33). Later, numerous other studies have reported AMIGO2 expression in gastrointestinal tract cancers, revealing its anti-apoptotic and cell adhesion activities which bestow to the tumor cells a higher metastasis formation capacity (34–38). Therefore, considering that cell adhesion and migration are two of the main mechanisms involved in the reorganization of the SV wall during the formation of the neointima, we explored the putative functions of AMIGO2 in mechanically stimulated SVPs and the signaling that may lie upstream of its differential expression at a transcriptional level.

**Figure 3:**
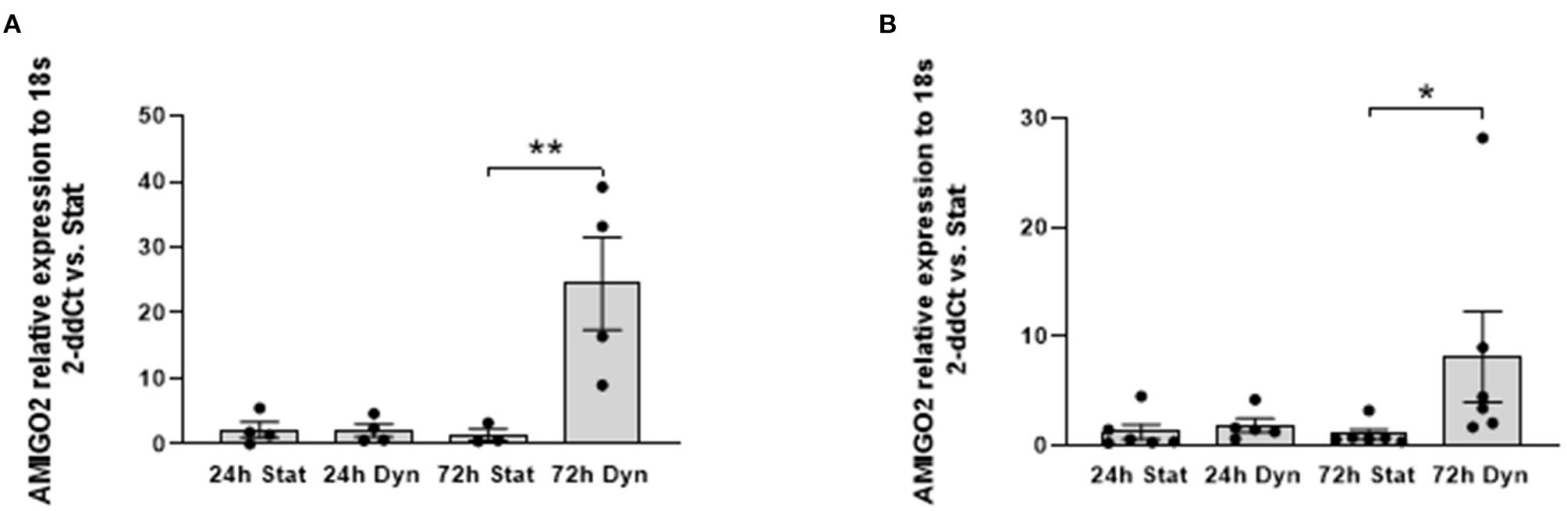
RNA-seq validation. Bar graphs showing relative expression of *AMIGO2* in SVPs after mechanical stimulation assessed by real-time qPCR. **(A)** Analysis performed in the same samples of the RNA-Seq (*N* = 5); difference among groups are evaluated using one-way ANOVA for normal distributed values, based on the result of normality tests. **(B)** Analysis performed using SVPs obtained from a new set of donors (*N* = 7), statistical analysis conducted using Kruskal–Wallis test for not normal distributed values, based on results from normality tests. Data are presented as mean ± SEM of 2-ddCt vs static (Stat) condition of each time point, *p < 0.05 **p < 0.01 (*t*-test stat vs. dyn).

First, we validated the RNA-Seq results using RT-qPCR on the same RNA pools used to perform the RNA-seq and on RNAs isolated from SVPs of different patients subjected to uniaxial strain. Results confirmed the overexpression of AMIGO2 in the SVPs when mechanically stimulated (Figure 3).

**Figure 4:**
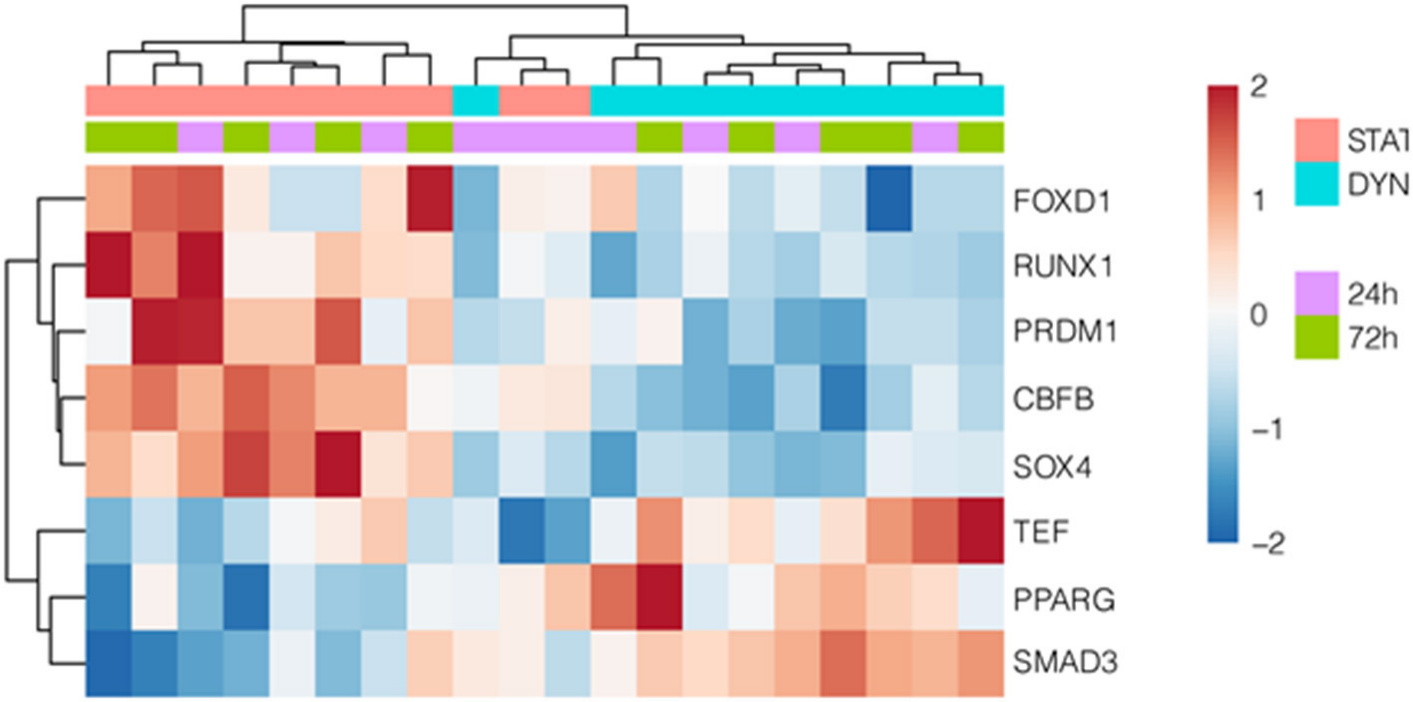
Transcription factors potentially regulating *AMIGO2*. Hierarchically clustered heatmap showing differentially expressed TFs, that are predicted to regulate AMIGO2. The motif-based TF prediction was performed using iRegulon on the complete set of DEGs at both time points.

**Figure 5:**
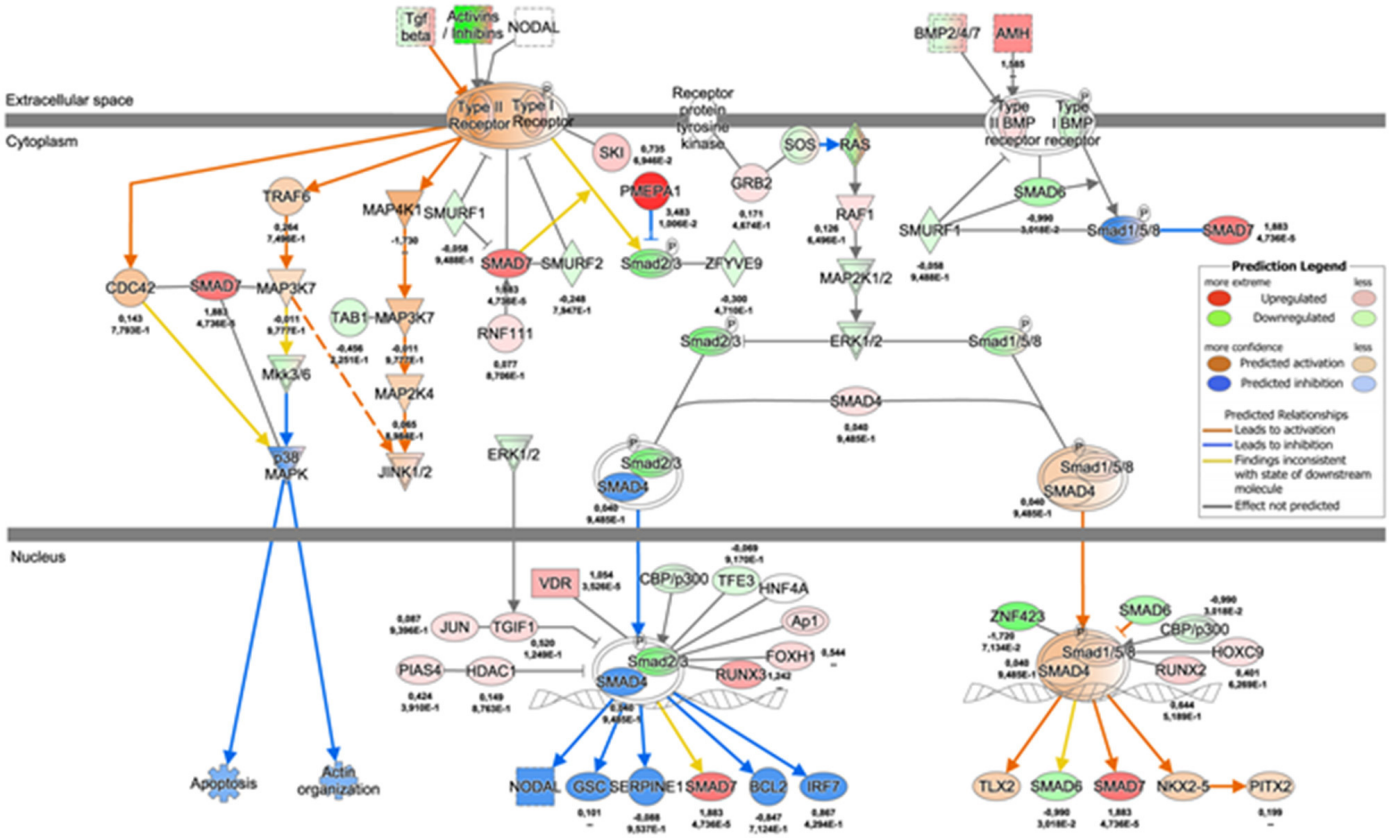
TGF-β signaling pathway map 72 h dyn vs. 72 h stat condition. Network depicting the regulation of TGF-β signaling cascade at 72 h, as represented by IPA. The values underneath each protein (when expressed in the dataset) indicate respectively the logFC and the adjusted *p*-value from the DE analysis between 72 h dyn and 72 h stat.

**Figure 6:**
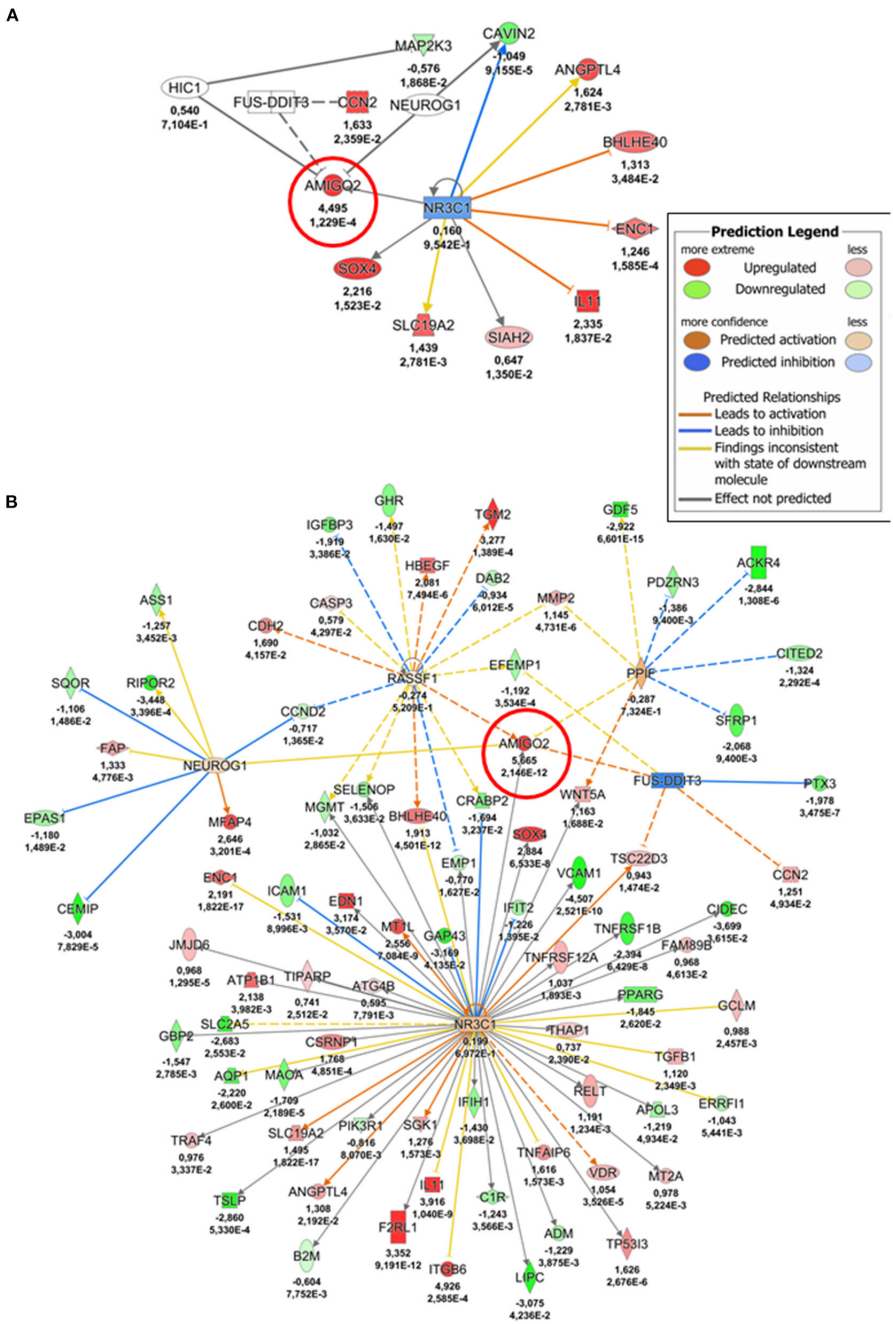
AMIGO2 upstream regulators-gene interaction networks. **(A)** Network depicting *AMIGO2* upstream regulators predicted activity at 24 h (24 h dyn vs. 24 h stat). **(B)** Network depicting *AMIGO2* upstream regulators predicted activity at 72 h (72 h dyn vs. 72 h stat). The values underneath each gene (when expressed in the dataset) indicate respectively the logFC and the adjusted *p*-value from the DE analysis. AMIGO2 is circled in red.

We then investigated the regulation of *AMIGO2* in SVPs under mechanical stress by examining the DEGs in all data sets, taking into consideration the AMIGO2-associated expression processes, the interactions with other proteins, and the biological functions in which it is putatively involved. First, through iRegulon motif-based prediction we identified 8 transcription factors (TFs) potentially involved in *AMIGO2* expression regulation. Hierarchical clustering showed a partial grouping among SVPs samples after 24 h of strain, which becomes robust when considering the samples cultured for 72 h (Figure 4). Interestingly, 5 out of 8 TFs were related to transforming growth factor β (TGF-β) pathways (*RUNX1, PRDM1, SOX4, PPARG*, and *SMAD3*), known for modulating mesenchymal phenotype acquisition (39–43). The TF *CBFB* acts in coordination with *RUNX1* regulating the transcription of several genes, one of which is *NOTCH3* (44). *FOXD1* mediates gene expression of the cell during the reprogramming process (45). These activities combined with the negative effect of stretching on *TEF* expression suggest the switching of SVPs toward a proliferative phenotype (46). For a better understanding the effects of the mechanical stretching on the TGF-β pathway, we performed Ingenuity Pathway Analysis (IPA) at both 24 h dyn vs. 24 h stat (Supplementary Figure 4) and 72 h dyn vs. 72 h stat conditions (Figure 5). Through pathway enrichment analysis for TGF-β signaling at 72 h dyn vs. 72 h stat, we were able to identify 36 genes associated with the TGF-β pathway that were down-regulated and 45 genes up-regulated (Supplementary Figure 5). To show relevant relationships between modulated genes we performed IPA analysis in the complete dataset. The topmost identified networks are shown in Figures 6A,B. In both comparisons 24 h dyn vs. 24 h stat and 72 h dyn vs. 72 h stat, *AMIGO2* expression was predicted to be controlled by *NR3C1*, the human glucocorticoid receptor gene, and *NEUROG1*, a regulator of neural progenitors' differentiation (47, 48). Connections were also found for the mesenchymal oncogenes *FUS-DDIT3* at both time points and for *RASSF1* at 72 h (49, 50).

### AMIGO2 Expression Is Associated With a Mechanical Strain-Mediated Phenotypic Shift in SVPs

**Figure 7:**
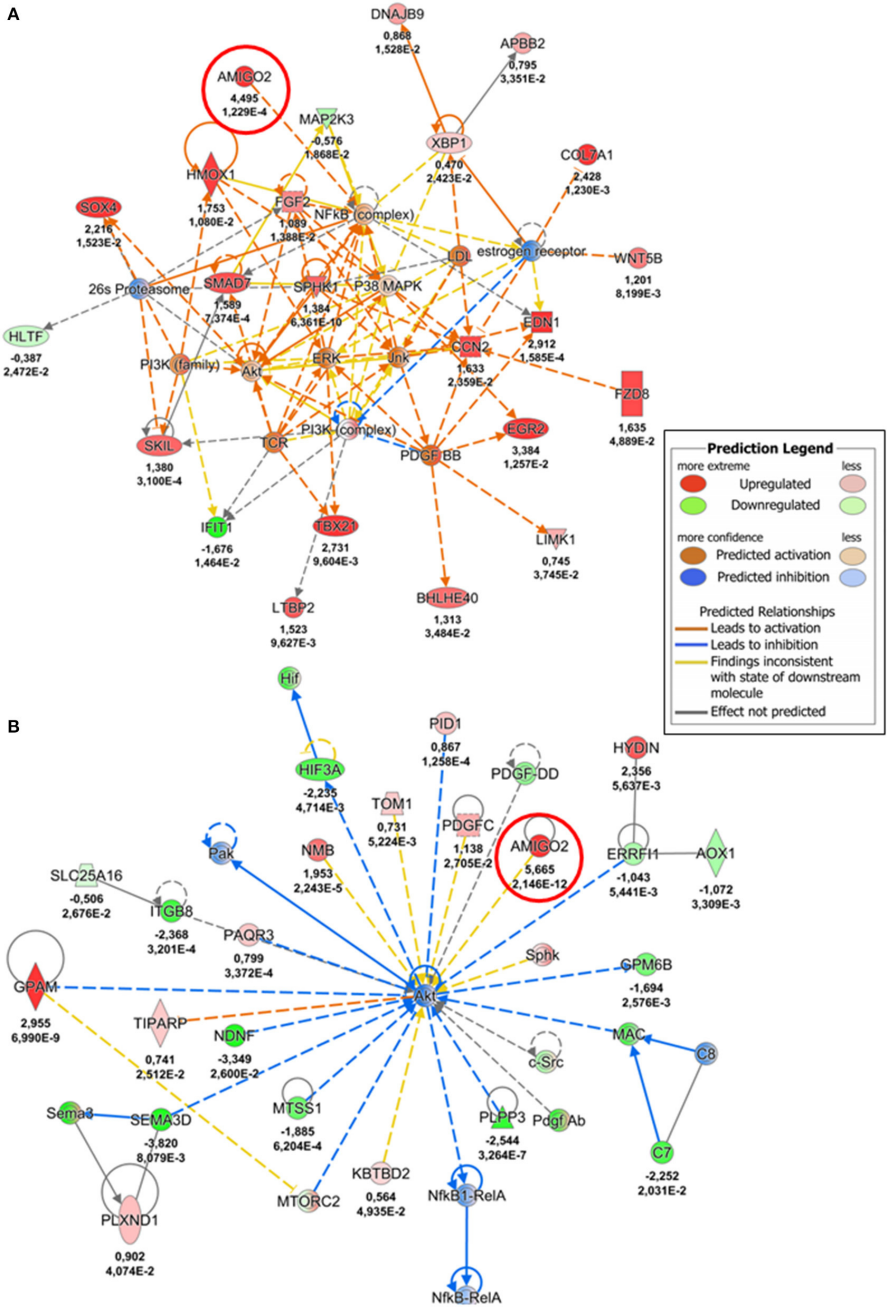
*AMIGO2-protein* interaction networks. **(A)** Protein-protein interaction network involving AMIGO2, identified by IPA analysis (24 h dyn vs. 24 h stat). **(B)** Protein-protein interaction network involving AMIGO2, identified by IPA analysis (72 h dyn vs. 72 h stat). The values underneath each protein (when expressed in the dataset) indicate respectively the logFC and the adjusted *p*-value from the DE analysis. AMIGO2 is circled in red.

**Figure 8:**
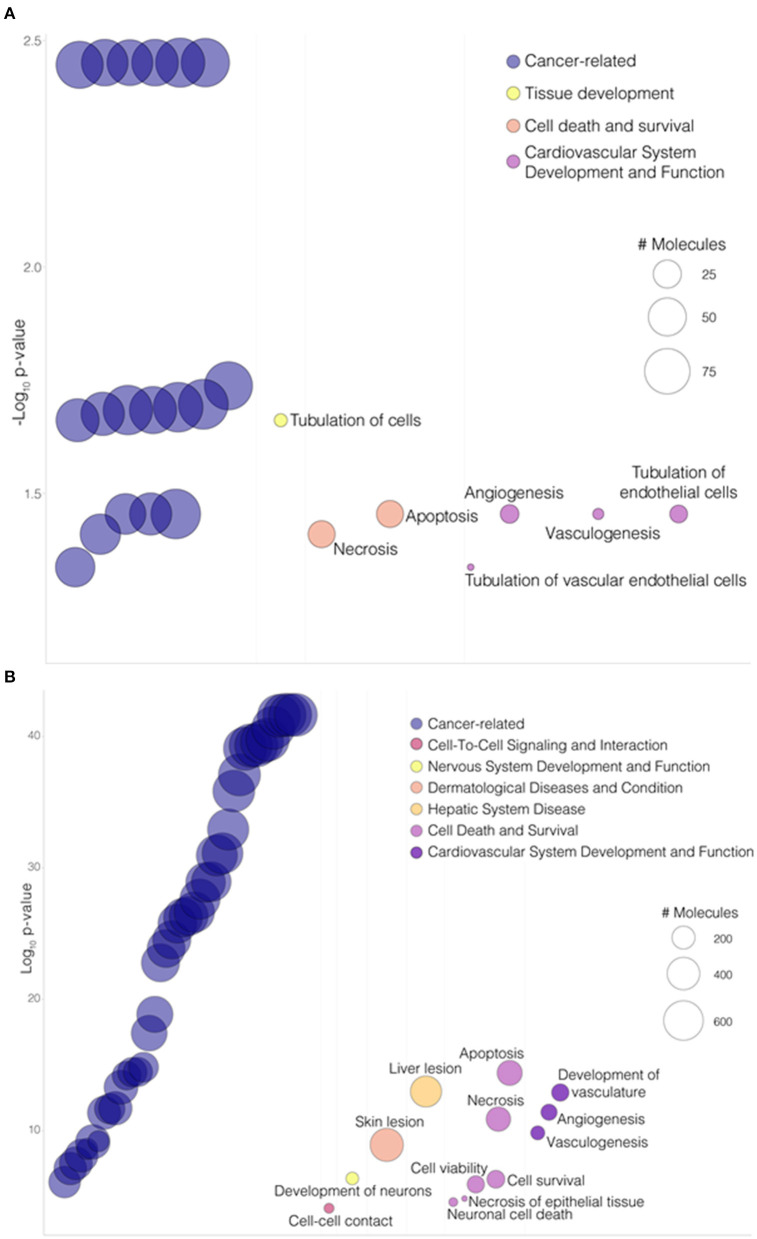
AMIGO2 associated functions. **(A)** Bubbleplot portraying a selection of “Diseases and Biological Functions” enriched pathways involving AMIGO2 from IPA analysis, in the 24 h dyn vs. 24 h stat comparison. **(B)** Bubbleplot portraying a selection of “Diseases and Biological Functions” enriched pathways involving AMIGO2 from IPA analysis, in the 72 h dyn vs. 72 h stat comparison. Pathways are organized by macro-categories on the x-axis and ordered by -log_10_BH-adjusted *p*-value on the y-axis. The bubble size is proportional to the number of molecules involved in the pathway.

To gain further insight into mechanical strain-associated changes in SVPs in relation to *AMIGO2* we analyzed the human protein-protein interaction networks by IPA (Figures 7A,B). In the 24 h dyn vs. 24 h stat comparison, AMIGO2 was found connected to NFkB complex which was predicted up-regulated by the modulation of DEGs present in the network. After 72 h of culture, the specific modulation of several genes, including AMIGO2, suggested an interaction with Akt, which was predicted to be inhibited as a result of the stretch-dependent changes in the transcriptional profile of SVPs. This finding was in contrast with what has been reported in AMIGO2's activation mechanism in endothelial cells (ECs) (36), and suggests that in SVPs AMIGO2 could operate through different pathways, independent of PDK-Akt. Next, the effect of mechanical strain on the molecular and cellular functions of SVPs has been closely examined via Gene Ontology (GO) analysis of the 24 h dyn vs. 24 h stat and 72 h dyn vs. 72 h stat conditions. Table 4 shows the top 5 enriched pathways involving AMIGO2. The topmost function in SVPs stretched cells was cell-matrix adhesion (*p*-value 7.72 × 10^−10^), along with chemotaxis (*p*-value 6.92 × 10^−10^) and cell-cell adhesion via plasma-membrane adhesion molecules (*p*-value 2.07 × 10^−10^), describing a potential switching of the SVPs phenotype toward migration. Ultimately, diseases and biological function-related pathways involving AMIGO2 were obtained through IPA (Figures 8A,B). We observed an increase in the number of functions identified prolonging mechanical stress from 24 to 72 h. For example, the Cell Death and Survival macro-category after 24 h addressed only the regulation of apoptosis and necrosis, but after 72 h we also detected activation of cell viability and cell survival pathways. Of note, the cancer-related biofunctions were the most associated with AMIGO2 at both time points, reflecting its potential role in tissue invasion and remodeling (34, 35, 37, 38). Thus, the investigations performed with iRegulon and IPA as well as the AMIGO2-related GO findings shed a light on how mechanical stress could produce a phenotypic switching in the SVPs, mediating their activation and increasing their responsiveness to modifications occurring in the extracellular environment.

**Table 4:** AMIGO2 related pathways regulation.

**Term**	**Description**	**–Log10 value**
GO:0098742	Cell-cell adhesion via plasma-membrane adhesion molecules	**2.07**
GO:0007160	Cell-matrix adhesion	**7.72**
GO:0044089	Positive regulation of cellular component biogenesis	**3.74**
GO:0006935	Chemotaxis	**6.92**
GO:0051962	Positive regulation of nervous system development	**4.88**

### Arterial-Like Mechanic of SV Induces an Increase of AMIGO2 Positive Cells Only in Long Term *in vivo* Model

**Figure 9:**
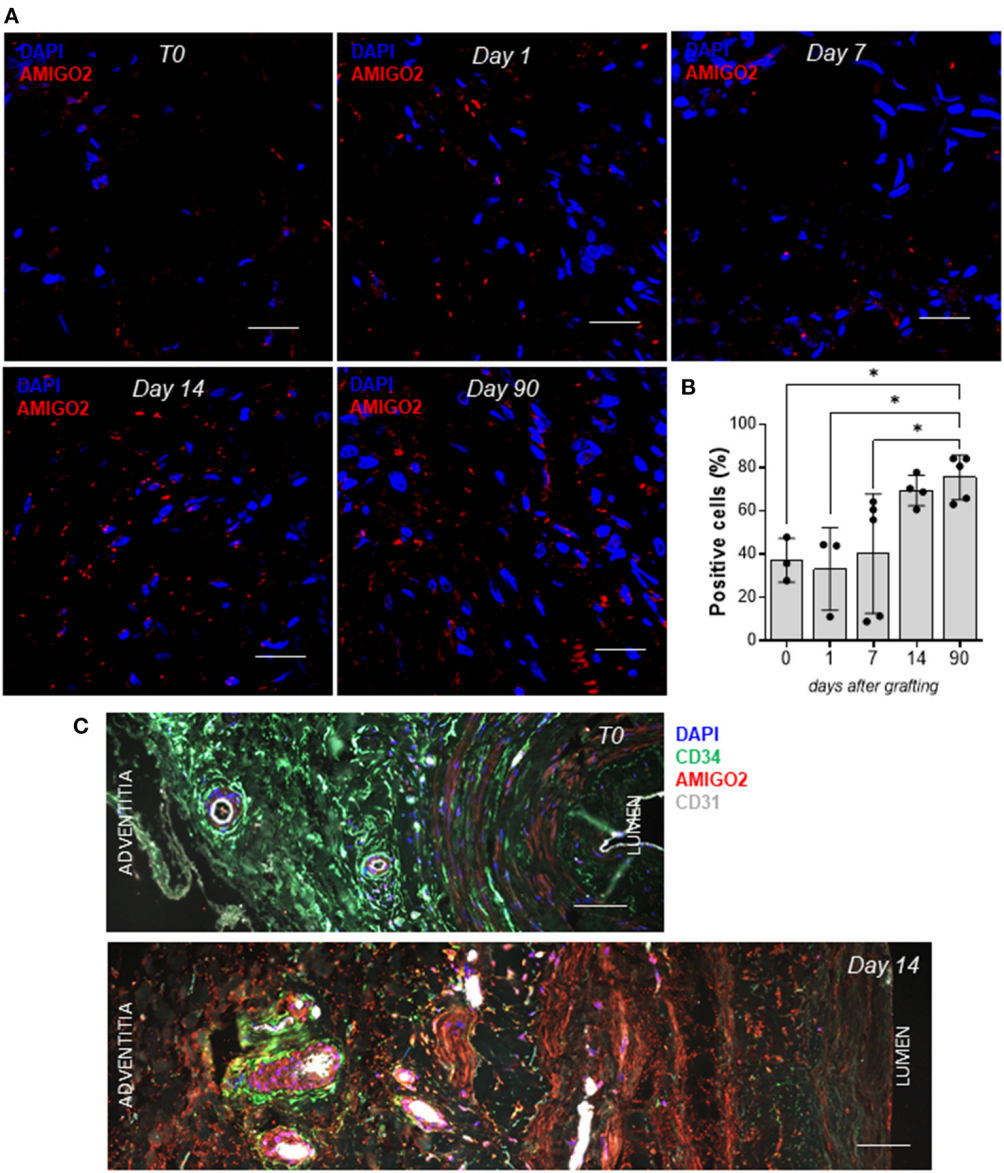
AMIGO2 expression in vein graft remodeling. **(A)** Confocal microscopy analysis of paraffin-embedded porcine SVs for AMIGO2 (red) expression, nuclei are labeled with DAPI. Scale bar indicates 20 μm. **(B)** Bar graphs showing the percentage of AMIGO2 positive cells in porcine SV adventitia after grafting into the carotid artery (T0: *N* = 3, Day 1: *N* = 3, Day 7: *N* = 5, Day 14: *N* = 4, Day 90: *N* = 5; all data shown as Mean ± SEM, * = *p* < 0.05). **(C)** Confocal microscopy analysis of paraffin-embedded human SVs for CD34 (green), AMIGO2 (red), CD31 (white), nuclei are labeled with DAPI. Scale bar indicates 50 μm.

Given that the cyclic mechanical stress on SVPs produced a significant overexpression of AMIGO2 at the transcription level, we explored the effect of pulsatile coronary flow on AMIGO2 protein expression. This was assessed in two experimental systems that we previously used to validate Thrombospondin-1 as a relevant target of mechanical stress in the human SV arterialization process (19). These consisted of an *in vivo* SV arterialization model, performed by surgical SV interposition into carotid arteries in pigs, and of direct stimulation of human SVs using a coronary pulse duplicator that allows reproducing the mechanical conditions of the coronary circulation *in vitro*. Immunofluorescence staining was used to quantify the percentage of AMIGO2 positive cells in pig SV native conduits (T0) and at 1, 7, 14, and 90 days after grafting into the carotid artery (Figure 9A). <40% of the cells in the SV adventitia expressed AMIGO2 at T0 (37.13 ± 5.84%, *N* = 3) (Figure 9B). This percentage remained relatively unaffected during the following 7 days after surgery. On day 14, the percentage reached almost 70% of the total cells of the adventitia (69.35 ± 3.52%, *N* = 4), but this was not statistically different from the previous time points. Only at day 90 post-surgery, we observed a significant increase of AMIGO2 positive cells compared to T0, day 1, and day 7. In addition, we successfully identified numerous AMIGO2 expressing cells in human SV, both in the untapped conduit (T0) and following the application of a pulsatile pressure regimen for 14 days in *ex vivo* culture (Figure 9C). It is interesting to note that in both experiments AMIGO2 expression was not restricted to the adventitia layer, but was also detected in several cells in the tunica media, as well as ECs. Particularly significant was the detection of AMIGO2/CD34 positive cells in the proximity of the *vasa vasorum*, where the SVPs are normally localized (21). The quantification of AMIGO2 expression in SVs from 4 different donors did not highlight any significant difference in the comparison T0 vs. Day 14 (data not shown). These data consolidate AMIGO2 as an important effector in the response to pathologic mechanical stress. Thus, despite the early AMIGO2 upregulation driven by the mechanical strain at 24 and 72 h in the SVPs, the protein increase in the adventitia appears to be delayed when the entire vein wall undergoes pulsatile arterial flow.

## Discussion

In this study, we showed for the first time that mechanical strain specifically alters the transcriptomic profile of human SVPs. The validation of the identified molecules and the further investigation of the molecular mechanisms associated with vein arterialization in specific cells is fundamental to developing targeted preventive and curative strategies to combat the failure of bypass grafts.

The existing causal relationship between arterial hemodynamic and IH in vein grafts is long known and well-established (51). Experimental evidence demonstrated that when dissected veins are re-anastomosed to venous circulation they do not develop IH (52), while vein grafts transposed from the arterial circulation back to the venous flow exhibited the regression of IH (53, 54). Therefore, mechanical stress resulting from the coronary flow pattern is sufficient for the molecular setting of IH. Further validation of the detrimental role of coronary mechanical load on the integrity of the SV wall is provided by the significant reduction in IH and beneficial effects on vessel compliance exerted by external stenting or photochemical tissue passivation (20, 55). Recently we demonstrated the role of mechanical forces in the pathologic evolution of the human SV, identifying phenotypic switching of resident smooth muscle cells and the activation of TGF-b/Thrombospondin-1 signaling in the SV medial layer as the nexus between the fibrotic activation of vessel-resident cells and non-physiologic vessel perfusion (19). We also demonstrated that coronary flow mechanics endured by the SV induces progressive recruitment of SVPs from the adventitia and transition toward the medial layer. In the current study, we assessed the mechanical sensitivity of the SVPs themselves which, like resident smooth muscle cells, were responsive to mechanical stress and thus may participate in the pathologic programming of the vein wall.

Using a platform that enables us to perform uniaxial strain *in vitro*, we subjected human primary culture amplified SVPs to a cyclic elongation pattern with a nominal deformation (10%) and a frequency (1 Hz) compatible with the predicted uniaxial strain component acting in SV wall (19). We then conducted a genome-wide RNA-Seq analysis to observe whether this stimulus was sufficient to elicit a robust change in SVPs gene expression. Through the analysis of DEGs in the four comparisons, two times of stimulation (24/72 h) and two experimental conditions (static/dynamic), we identified the 72 h dyn vs. 72 h stat as the most powerful in terms of transcriptomic alterations, with 819 DEGs. Interestingly, the variation in the transcriptome of SVPs subjected to mechanical stimulation occurred progressively and led to the identification of a few transcripts that were robustly up/downmodulated by mechanical treatment, highlighting them as “top scores” in the differentially expressed genes with possible important roles in SV pathologic programming.

Interestingly, this transcriptomic alteration is SVP-specific and potentially occurs before the phenotypic changes induced by paracrine signaling (17, 19). Moreover, SVP response to mechanical strain is similar to what has been observed in smooth muscle cells and ECs when comparable mechanical stress was applied (56). Several studies have demonstrated that cyclic strain exerts an effect on cellular proliferation, but there is no agreement with regard of this effect increases or reduces cell mitosis (56). Our results showed the activation in SVPs of apoptosis and necrosis pathways after 24 h with the addition of necrosis of epithelial tissue, neuronal cell death, cell viability, and cell survival pathways after 72 h of mechanical stress. This rather conflicting data would require further analysis. However, in the physiological environment of SV subjected to arterial flow, the paracrine signaling seems to generate a positive effect on SVPs proliferation, mainly through TGF-β and TSP-1 (19).

Among the DEGs, we found particularly interesting the upregulation of *AMIGO2* for its putative role in intercellular communication and cell migratory activity, one of the functions which appear to be activated in SVPs by mechanical straining. AMIGO2 was first described in the development and survival of the nervous system, although following studies discovered numerous activities related to its expression in different cell phenotypes (33–38). AMIGO2 belongs to the leucine-rich repeat (LRR) protein superfamily. LRR proteins share a common structural framework of 20 to 30 amino acids rich in the hydrophobic amino acid leucine (57). This family includes intracellular, extracellular, and membrane proteins with a wide range of functions such as cell adhesion, signaling, extracellular matrix assembly, RNA processing, and immune response. AMIGO2 acts as a cell adhesion molecule involved in signal transduction and, like other LRR proteins, functions mainly through homophilic and heterophilic interactions with proteins of the same family, i.e. AMIGO and AMIGO3 (32, 34). It has been reported that in ECs and in gastric adenocarcinoma cell lines the inhibition of AMIGO2 affected the ability to adhere to extracellular matrix components (34, 36). Moreover, Hossain et al. demonstrated the presence of *AMIGO2* in human microvascular ECs and pericytes, pointing to an interaction between these cells in vascular remodeling (58). In mechanically stressed SVPs the up-regulation of *AMIGO2* could confer a firmer adhesion to adhesion substrates but also enhance their sensitivity to modifications of the extracellular matrix. Since mechanical strain during IH induces the remodeling of LLR proteins (such as biglycan, versican, and decorin) (59–62), AMIGO2 could participate in SVP migration across the SV wall as observed in our previous study. This hypothesis is further supported by our GO analysis performed to identify the top regulated molecular functions enriched with AMIGO2 showing that in stretched SVPs cell-matrix adhesion, chemotaxis, and cell-cell adhesion via plasma-membrane adhesion molecules pathways were significantly up-regulated compared to static cultures, and by our unpublished evidences showing that mechanically strained-SVPs have a higher migratory mobility (Garoffolo et al., in preparation). Moreover, this finding is coherent to the invasive behavior that *AMIGO2* expression provides to tumor cells in the formation of metastasis (34, 35, 37, 38). Furthermore, a protein belonging to the LLR family, TSP-1, was already described as SVP's migration drive r (19). Notably, motif-based prediction of TFs potentially regulating *AMIGO2* revealed the presence of 5 TFs involved in TGF-β pathway. Previous studies reported that arterial-mimicking pressure elevates the expression of TGF-β in the SV and that TGF-β directly influences both ECs and SVPs, increasing the expression of SMC/mesenchymal differentiation markers and proliferation (19, 63). In addition, the presence of AMIGO2 positive cells in the tunica media and not only the adventitia of the *ex vivo* stimulated human SVs and of *in vivo* arterialized pig SVs strongly suggest the involvement of the protein in a fibrotic process of the vessel wall controlled by mechanical-dependent pathways. Finally, we believe it would be worthy to investigate if the proneuronal transcription factor NEUROG1 (64) and the glucocorticoid receptor NR3C1 (48) play a role in the SVPs differentiation toward intimal hyperplasia onset in the contest of mechanical stress.

In summary, our results concur to a better understanding of the mechanisms underlying the SV remodeling and reveal a novel target for future investigations. In particular, new studies are warranted to assess the regulation of *AMIGO2* within the combination of mechanical and paracrine stimuli, such as TGF-β, to support the relevance of mechanically activated pathways in the onset and the progression of vein graft disease.

## Data Availability Statement

The datasets presented in this study can be found in online repositories. The names of the repository/repositories and accession number(s) can be found below: https://www.ncbi.nlm.nih.gov/, GSE192712.

## Ethics Statement

This study was reviewed and approved by the Local Ethical Committee at Centro Cardiologico Monzino, IRCCS. All subjects gave their written informed consent to participate. The animal study was performed in accordance with the Home Office Guidance on the Operation of the Animals (Scientific Procedures) Act 1986 (HMSO, London, UK; PPL numbers 30/2585 and 30/3064) and was compliant with the EU Directive 2020/63/EU and principles stated in the Guide for the Care and Use of Laboratory Animals (Institute of Laboratory Animal Resources, 1996).

## Author Contributions

DM, GG, AT, MR, and RV performed experiments and analyzed data. GC analyzed data. MP, PM, and GS conceived the study. DM and GS wrote the paper. All authors contributed to the article and approved the submitted version.

## Funding

Funding/financial support was obtained from the Italian Ministry of Health, Ricerca Corrente to the IRCCS MultiMedica and Ricerca Finalizzata 2011 (project code: RF-2011-02346867) and was also supported by the Heart Research UK grant ‘Targeting pericytes for halting pulmonary hypertension in infants with congenital heart disease’ (R102602).

## Conflict of Interest

The authors declare that the research was conducted in the absence of any commercial or financial relationships that could be construed as a potential conflict of interest.

## Publisher's Note

All claims expressed in this article are solely those of the authors and do not necessarily represent those of their affiliated organizations, or those of the publisher, the editors and the reviewers. Any product that may be evaluated in this article, or claim that may be made by its manufacturer, is not guaranteed or endorsed by the publisher.

## References

[1] Head SJ Milojevic M Taggart DP Puskas JD. Current practice of state-of-the-art surgical coronary revascularization. Circulation. (2017) 136:1331–45. 10.1161/CIRCULATIONAHA.116.022572 28972063

[2] Raja SG Haider Z Ahmad M Zaman H. Saphenous vein grafts: To use or not to use? Hear Lung Circ. (2004) 13:150–6. 10.1016/j.hlc.2004.03.013 16352186

[3] Osgood MJ Hocking KM Voskresensky I V Li FD Komalavilas P Cheung-Flynn J Brophy CM. Surgical vein graft preparation promotes cellular dysfunction, oxidative stress, and intimal hyperplasia in human saphenous vein. J Vasc Surg. (2014). 60: 202–11. 10.1016/j.jvs.2013.06.004 23911244 PMC3926896

[4] Locker C Schaff H V Dearani JA Joyce LD Park SJ Burkhart HM. Multiple arterial grafts improve late survival of patients undergoing coronary artery bypass graft surgery: analysis of 8,622 patients with multivessel disease. Circulation. (2012) 126:1023–30. 10.1161/CIRCULATIONAHA.111.084624 22811577

[5] Shukla N Jeremy JY. Pathophysiology of saphenous vein graft failure: A brief overview of interventions. Curr Opin Pharmacol. (2012) 12:114–20. 10.1016/j.coph.2012.01.001 22321569

[6] Wallitt EJW Jevon M Hornick PI. Therapeutics of Vein Graft Intimal Hyperplasia: 100 Years On. Ann Thorac Surg. (2007) 84:317–23. 10.1016/j.athoracsur.2007.02.035 17588453

[7] Dashwood MR Tsui JC. “No-touch” saphenous vein harvesting improves graft performance in patients undergoing coronary artery bypass surgery: a journey from bedside to bench. Vascul Pharmacol. (2013) 58:240–50. 10.1016/j.vph.2012.07.008 22967905

[8] Davies MG Hagen PO. Reprinted Article “pathophysiology of vein graft failure: a review.” *Eur J Vasc Endovasc Surg*. (2011) 42. S19–29. 10.1016/j.ejvs.2011.06.013 21855014

[9] Bouten CVC Dankers PYW Driessen-Mol A Pedron S Brizard AMA Baaijens FPT. Substrates for cardiovascular tissue engineering. Adv Drug Deliv Rev. (2011) 63:221–41. 10.1016/j.addr.2011.01.007 21277921

[10] Malek AM Alper SL Izumo S. Hemodynamic shear stress and its role in atherosclerosis. J Am Med Assoc. (1999) 282:2035–42. 10.1001/jama.282.21.2035 10591386

[11] Davies MG Klyachkin ML Dalen H Massey MF Svendsen E Hagen PO. The integrity of experimental vein graft endothelium-implications on the etiology of early graft failure. Eur J Vasc Surg. (1993) 7:156–65. 10.1016/S0950-821X(05)80756-X 8462704

[12] Tai NR Salacinski HJ Edwards A Hamilton G Seifalian AM. Compliance properties of conduits used in vascular reconstruction. Br J Surg. (2000) 87:1516–24. 10.1046/j.1365-2168.2000.01566.x 11091239

[13] Fry DL. Acute vascular endothelial changes associated with increased blood velocity gradients. Circ Res. (1968) 22:165–97. 10.1161/01.RES.22.2.165 5639037

[14] Newby AC Zaltsman AB. Molecular mechanisms in intimal hyperplasia. J Pathol. (2000) 190:300–9. 10.1002/(SICI)1096-9896(200002)190:3<300::AID-PATH596>3.0.CO;2-I 10685064

[15] Ward AO Caputo M Angelini GD George SJ Zakkar M. Activation and inflammation of the venous endothelium in vein graft disease. Atherosclerosis. (2017) 265:266–74. 10.1016/j.atherosclerosis.2017.08.023 28865843

[16] O'Callaghan CJ Williams B. Mechanical strain-induced extracellular matrix production by human vascular smooth muscle cells: role of TGF-β1. Hypertension. (2000) 36:319–24. 10.1161/01.HYP.36.3.319 10988258

[17] Prandi F Piola M Soncini M Colussi C D'Alessandra Y Penza E. Adventitial vessel growth and progenitor cells activation in an ex vivo culture system mimicking human saphenous vein wall strain after coronary artery bypass grafting. PLoS ONE. (2015) 10:e0117409. 10.1371/journal.pone.0117409 25689822 PMC4331547

[18] McGeachie J Campbell P Prendergast F. Vein to artery grafts. A quantitative study of revascularization by vasa vasorum and its relationship to intimal hyperplasia. Ann Surg. (1981) 194:100–7. 10.1097/00000658-198107000-00018 7247528 PMC1345203

[19] Garoffolo G Ruiter MS Piola M Brioschi M Thomas AC Agrifoglio M. Coronary artery mechanics induces human saphenous vein remodelling via recruitment of adventitial myofibroblast-like cells mediated by thrombospondin-1. Theranostics. (2020) 10:2597–611. 10.7150/thno.40595 32194822 PMC7052885

[20] Salinas HM Khan SI McCormack MC Fernandes JR Gfrerer L Watkins MT Redmond RW Austen WG. Prevention of vein graft intimal hyperplasia with photochemical tissue passivation. J Vasc Surg. (2017) 65:190–196. 10.1016/j.jvs.2015.11.049 27066947

[21] Campagnolo P Cesselli D Al Haj Zen A Beltrami AP Kränkel N Katare R. Human adult vena saphena contains perivascular progenitor cells endowed with clonogenic and proangiogenic potential. Circulation. (2010) 121:1735–45. 10.1161/CIRCULATIONAHA.109.899252 20368523 PMC2917746

[22] Edgar R Domrachev M Lash AE. Gene expression omnibus: NCBI gene expression and hybridization array data repository. Nucleic Acids Res. (2002) 30:207–10. 10.1093/nar/30.1.207 11752295 PMC99122

[23] Ewels P Magnusson M Lundin S Käller M. MultiQC: Summarize analysis results for multiple tools and samples in a single report. Bioinformatics. (2016) 32:3047–8. 10.1093/bioinformatics/btw354 27312411 PMC5039924

[24] Love MI Huber W Anders S. Moderated estimation of fold change and dispersion for RNA-seq data with DESeq2. Genome Biol. (2014) 15:550. 10.1186/s13059-014-0550-8 25516281 PMC4302049

[25] Janky R Verfaillie A Imrichová H van de Sande B Standaert L Christiaens V. iRegulon: From a Gene List to a Gene Regulatory Network Using Large Motif and Track Collections. PLoS Comput Biol. (2014) 10:e1003731. 10.1371/journal.pcbi.1003731 25058159 PMC4109854

[26] Krämer A Green J Pollard J Tugendreich S. Causal analysis approaches in ingenuity pathway analysis. Bioinformatics. (2014) 30:523–30. 10.1093/bioinformatics/btt703 24336805 PMC3928520

[27] Zhou Y Zhou B Pache L Chang M Khodabakhshi AH Tanaseichuk O. Metascape provides a biologist-oriented resource for the analysis of systems-level datasets. Nat Commun. (2019) 10:1–10. 10.1038/s41467-019-09234-6 30944313 PMC6447622

[28] Angelini GD Bryan AJ Williams HMJ Morgan R Newby AC. Distention promotes platelet and leukocyte adhesion and reduces short-term patency in pig arteriovenous bypass grafts. J Thorac Cardiovasc Surg. (1990) 99:433–9. 10.1016/S0022-5223(19)36973-9 2308361

[29] Thomas AC Wyatt MJ Newby AC. Reduction of early vein graft thrombosis by tissue plasminogen activator gene transfer. Thromb Haemost. (2009) 102:145–52. 10.1160/TH08-11-0772 19572079

[30] Piola M Ruiter M Vismara R Mastrullo V Agrifoglio M Zanobini M. Full mimicking of coronary hemodynamics for ex-vivo stimulation of human saphenous veins. Ann Biomed Eng. (2017) 45:884–97. 10.1007/s10439-016-1747-7 27752921

[31] Piola M Prandi F Bono N Soncini M Penza E Agrifoglio M. compact and automated ex vivo vessel culture system for the pulsatile pressure conditioning of human saphenous veins. J Tissue Eng Regen Med. (2016) 10:E204–15. 10.1002/term.1798 23897837

[32] Kuja-Panula J Kiiltomäki M Yamashiro T Rouhiainen A Rauvala H. AMIGO a transmembrane protein implicated in axon tract development, defines a novel protein family with leucine-rich repeats. J Cell Biol. (2003) 160:963–73. 10.1083/jcb.200209074 12629050 PMC2173769

[33] Ono T Sekino-Suzuki N Kikkawa Y Yonekawa H Kawashima S. Alivin 1, a novel neuronal activity-dependent gene, inhibits apoptosis and promotes survival of cerebellar granule neurons. J Neurosci. (2003) 23:5887–96. 10.1523/JNEUROSCI.23-13-05887.2003 12843293 PMC6741272

[34] Rabenau KE O'Toole JM Bassi R Kotanides H Witte L Ludwig DL. DEGA/AMIGO-2, a leucine-rich repeat family member, differentially expressed in human gastric adenocarcinoma: effects on ploidy, chromosomal stability, cell adhesion/migration and tumorigenicity. Oncogene. (2004) 23:5056–67. 10.1038/sj.onc.1207681 15107827

[35] Tsoi LC Qin T Slate EH Zheng WJ. Consistent Differential Expression Pattern (CDEP) on microarray to identify genes related to metastatic behavior. Acta Vet Scand. (2011) 53:438. 10.1186/1471-2105-12-438 22078224 PMC3251006

[36] Park H Lee S Shrestha P Kim J Park JA Ko Y. AMIGO2, a novel membrane anchor of PDK1, controls cell survival and angiogenesis via Akt activation. J Cell Biol. (2015) 211:619–37. 10.1083/jcb.201503113 26553931 PMC4639856

[37] Kanda Y Osaki M Onuma K Sonoda A Kobayashi M Hamada J. Amigo2-upregulation in tumour cells facilitates their attachment to liver endothelial cells resulting in liver metastases. Sci Rep. (2017) 7:1–13. 10.1038/srep43567 28272394 PMC5341090

[38] Nakamura S Kanda M Shimizu D Tanaka C Inokawa Y Hattori N. AMIGO2 expression as a potential prognostic biomarker for gastric cancer. Anticancer Res. (2020) 40:6713–21. 10.21873/anticanres.14694 33288564

[39] Kim W Barron DA Martin RS Chan KS Tran LL Yang F. RUNX1 is essential for mesenchymal stem cell proliferation and myofibroblast differentiation. Proc Natl Acad Sci U S A. (2014) 111:13389–6394. 10.1073/pnas.1407097111 25313057 PMC4246299

[40] Romagnoli M Belguise K Yu Z Wang X Landesman-Bollag E Seldin DC. Epithelial-to-mesenchymal transition induced by TGF-β1 is mediated by blimp-1-dependent repression of BMP-5. Cancer Res. (2012) 72:6268–78. 10.1158/0008-5472.CAN-12-2270 23054396 PMC3513653

[41] Peng X Liu G Peng H Chen A Zha L Wang Z. SOX4 contributes to TGF-β-induced epithelial–mesenchymal transition and stem cell characteristics of gastric cancer cells. Genes Dis. (2018) 5:49–61. 10.1016/j.gendis.2017.12.005 30258935 PMC6147107

[42] Kim SG Kim HA Jong HS Park JH Kim NK Hong SH. The endogenous ratio of Smad2 and Smad3 influences the cytostatic function of Smad3. Mol Biol Cell. (2005) 16:4672–83. 10.1091/mbc.e05-01-0054 16093355 PMC1237073

[43] Reka AK Kurapati H Narala VR Bommer G Chen J Standiford TJ. Peroxisome proliferator-activated receptor-g activation inhibits tumor metastasis by antagonizing smad3-mediated epithelial-mesenchymal transition. Mol Cancer Ther. (2010) 9:3221–32. 10.1158/1535-7163.MCT-10-0570 21159608 PMC3044476

[44] Malik N Yan H Moshkovich N Palangat M Yang H Sanchez V. The transcription factor CBFB suppresses breast cancer through orchestrating translation and transcription. Nat Commun. (2019) 10:1–15. 10.1038/s41467-019-10102-6 31061501 PMC6502810

[45] Koga M Matsuda M Kawamura T Sogo T Shigeno A Nishida E. Foxd1 is a mediator and indicator of the cell reprogramming process. Nat Commun. (2014) 5:1–9. 10.1038/ncomms4197 24496101

[46] Yang J Wang B Chen H Chen X Li J Chen Y Yuan D Zheng S. Thyrotroph embryonic factor is downregulated in bladder cancer and suppresses proliferation and tumorigenesis via the AKT/FOXOs signalling pathway. Cell Prolif. (2019) 52: e12560. 10.1111/cpr.12560 30515906 PMC6496933

[47] Palma-Gudiel H Córdova-Palomera A Leza JC Fañanás L. Glucocorticoid receptor gene (NR3C1) methylation processes as mediators of early adversity in stress-related disorders causality: a critical review. Neurosci Biobehav Rev. (2015) 55:520–35. 10.1016/j.neubiorev.2015.05.016 26073068

[48] MuhChyi C Juliandi B Matsuda T Nakashima K. Epigenetic regulation of neural stem cell fate during corticogenesis. Int J Dev Neurosci. (2013) 31:424–33. 10.1016/j.ijdevneu.2013.02.006 23466416

[49] Bennani-Baiti IM. Epigenetic and epigenomic mechanisms shape sarcoma and other mesenchymal tumor pathogenesis. Epigenomics. (2011) 3:715–32. 10.2217/epi.11.93 22126291

[50] Pérez-Mancera PA Sánchez-García I. Understanding mesenchymal cancer: The liposarcoma-associated FUS-DDIT3 fusion gene as a model. Semin Cancer Biol. (2005) 15:206–14. 10.1016/j.semcancer.2005.01.006 15826835

[51] Owens CD. Adaptive changes in autogenous vein grafts for arterial reconstruction: Clinical implications. J Vasc Surg. (2010) 51:736–46. 10.1016/j.jvs.2009.07.102 19837532 PMC2834835

[52] Brody WR Angeli WW Kosek JC. Histologic fate of the venous coronary artery bypass in dogs. Am J Pathol. (1972) 66:111–30. 5009248 PMC2032468

[53] Davies MG Fulton GJ Svendsen E Hagen PO. Time course of the regression of intimal hyperplasia in experimental vein grafts. Cardiovasc Pathol. (1999) 8:161–8. 10.1016/S1054-8807(98)00029-5 10722239

[54] Fann JI Sokoloff MH Sarris GE Yun KL Kosek JC Miller DC. The reversibility of canine vein-graft arterialization. Circulation. (1990) 82:IV9–18. 2225440

[55] Moodley L Franz T Human P Wolf MF Bezuidenhout D Scherman J. Protective constriction of coronary vein grafts with knitted nitinol. Eur J Cardio-thoracic Surg. (2013) 44:64–71. 10.1093/ejcts/ezs670 23295444 PMC3708718

[56] Riha GM Lin PH Lumsden AB Yao Q Chen C. Roles of hemodynamic forces in vascular cell differentiation. Ann Biomed Eng. (2005) 33:772–9. 10.1007/s10439-005-3310-9 16078617

[57] Bella J Hindle KL McEwan PA Lovell SC. The leucine-rich repeat structure. Cell Mol Life Sci. (2008) 65:2307–33. 10.1007/s00018-008-8019-0 18408889 PMC11131621

[58] Hossain S Ahmed MU Alam S Watanabe A Harashima A Yonekura H Yamamoto H. Expressions and roles of AMIGO gene family in vascular endothelial cells. Int J Biosci Biochem Bioinforma. (2012) 10:1–5. 10.7763/IJBBB.2012.V2.58

[59] Scott L Kerr A Haydock D Merrilees M. Subendothelial proteoglycan synthesis and transforming growth factor beta distribution correlate with susceptibility to atherosclerosis. J Vasc Res. (1997) 34:365–77. 10.1159/000159245 9349730

[60] Hocking AM Shinomura T McQuillan DJ. Leucine-rich repeat glycoproteins of the extracellular matrix. Matrix Biol. (1998) 17:1–19. 10.1016/S0945-053X(98)90121-4 9628249

[61] Lee RT Yamamoto C Feng Y Potter-Perigo S Briggs WH Landschulz KT. Mechanical strain induces specific changes in the synthesis and organization of proteoglycans by vascular smooth muscle cells. J Biol Chem. (2001) 276:13847–51. 10.1074/jbc.M010556200 11278699

[62] Kenagy RD Kikuchi S Evanko SP Ruiter MS Piola M Longchamp A. Versican is differentially regulated in the adventitial and medial layers of human vein grafts. PLoS ONE. (2018) 13:e0204045. 10.1371/journal.pone.0204045 30265729 PMC6161854

[63] Cooley BC Nevado J Mellad J Yang D St Hilaire C Negro A Fang F Chen G San H Walts AD. TGF-β signaling mediates endothelial-to-mesenchymal transition (EndMT) during vein graft remodeling. Sci Transl Med. (2014) 6:227ra34. 10.1126/scitranslmed.3006927 24622514 PMC4181409

[64] Sun Y Nadal-Vicens M Misono S Lin MZ Zubiaga A Hua X. Neurogenin promotes neurogenesis and inhibits glial differentiation by independent mechanisms. Cell. (2001) 104:365–76. 10.1016/s0092-8674(01)00224-0 11239394

