# Supplementary material for: Mechanical Strain Induces Transcriptomic Reprogramming of Saphenous Vein Progenitors
Source: Front Cardiovasc Med. 2022 May 27;9:884031. doi: 10.3389/fcvm.2022.884031 (PMC9197233; doi:10.3389/fcvm.2022.884031)

Supplementary Material

Mechanical Strain Induces Transcriptomic Reprogramming of Saphenous Vein Progenitors

**Davide Maselli^1,2†^, Gloria Garoffolo^3†^, Giada Andrea Cassanmagnago^4,5^, Rosa Vono^1^, Matthijs S. Ruiter^3^, Anita C. Thomas^2^, Paolo Madeddu^2^, Maurizio Pesce^3§^, and Gaia Spinetti^1§*^.**

^1^IRCCS MultiMedica, Milan, Italy.

^2^University of Bristol, Bristol, United Kingdom.

*^3^Unità di Ingegneria Tissutale Cardiovascolare, Centro Cardiologico Monzino IRCCS, Milan, Italy.*

*^4^IRCCS Humanitas Research Hospital, Rozzano (MI), Italy.*

*^5^Department of Biomedical Sciences, Humanitas University, Pieve Emanuele (MI), Italy.*

***^†^*** These authors have contributed equally to this work and share first authorship

§ Co-senior authors

Supplementary Figures


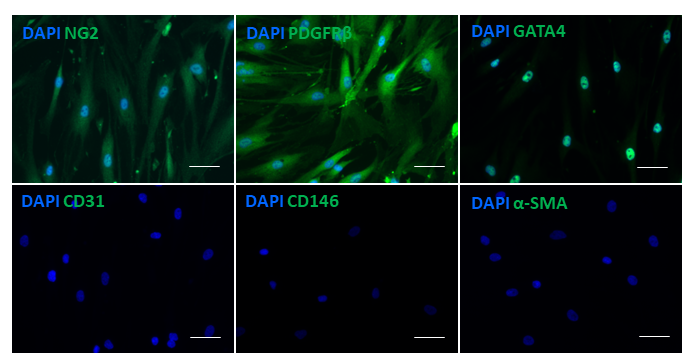
**Supplementary Figure 1: Immunocytochemical analysis of SVPs.** Characterization of the CD31^negative^/CD34^positive^ cell population for the expression of the SVPs markers: NG2, PDGFRβ and GATA4. Cells are negative for CD31, CD146 and α-SMA. Nuclei are labelled with DAPI. Scale bar indicates 50µm.

**Supplementary Figure 2: Cells under mechanical stimulation.** (A) Representative images of static SVPs culture after 72 hours**,** (B) and cells from the same donor subjected to uniaxial cyclic deformation protocol (0-10% deformation, 1 Hz frequency) for 72 hours. Cells are labeled
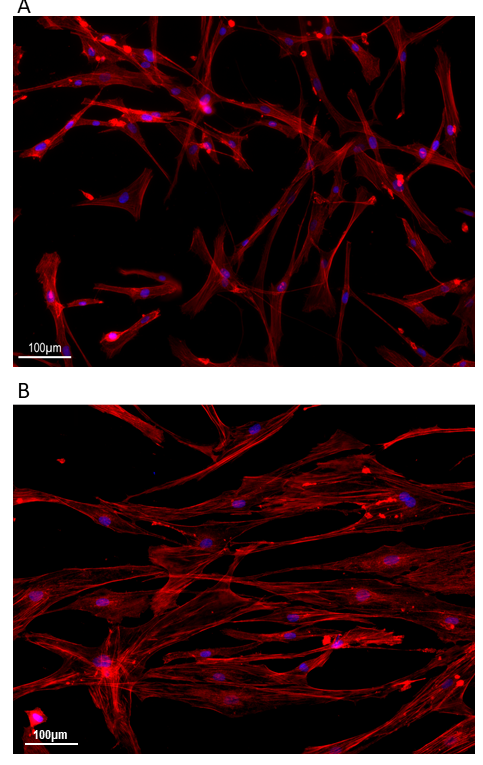
with Phalloidin-TRITC (red) and nuclei are stained with DAPI.


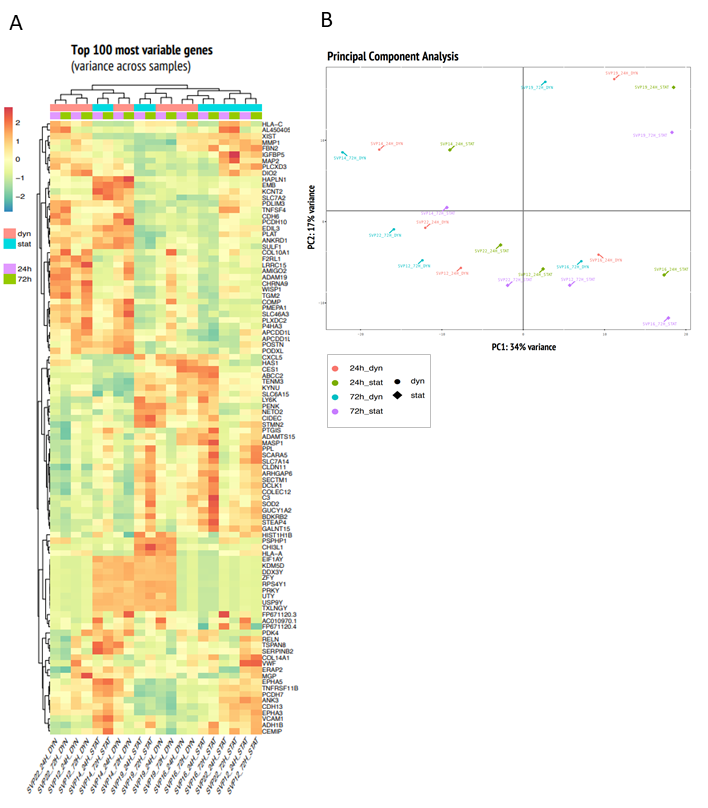


**Supplementary Figure 3: Assessment of sample variance across the entire dataset**. (A) Hierarchically clustered heatmap showing top 100 most variable genes across samples. (B) PC analysis was performed on all 20 samples.


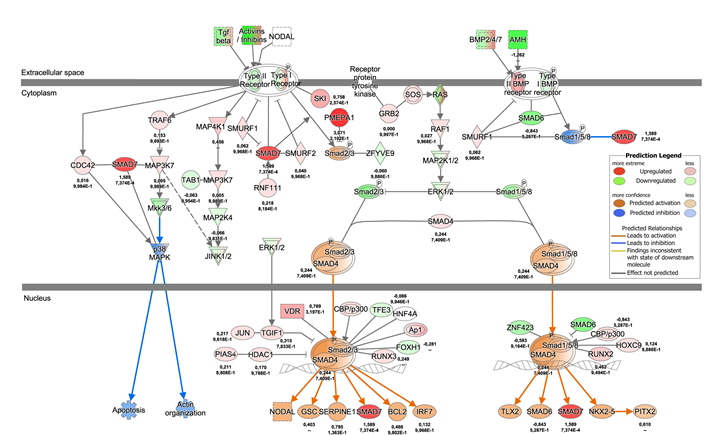
**Supplementary Figure 4: TGF-β signaling pathway map 24h dyn vs. 24h stat condition.** Network depicting the regulation of TGF-β signaling cascade at 24h, as represented by IPA. The values underneath each protein (when expressed in the dataset) indicate respectively the logFC and the adjusted p-value from the DE analysis between 24h dyn and 24h stat.


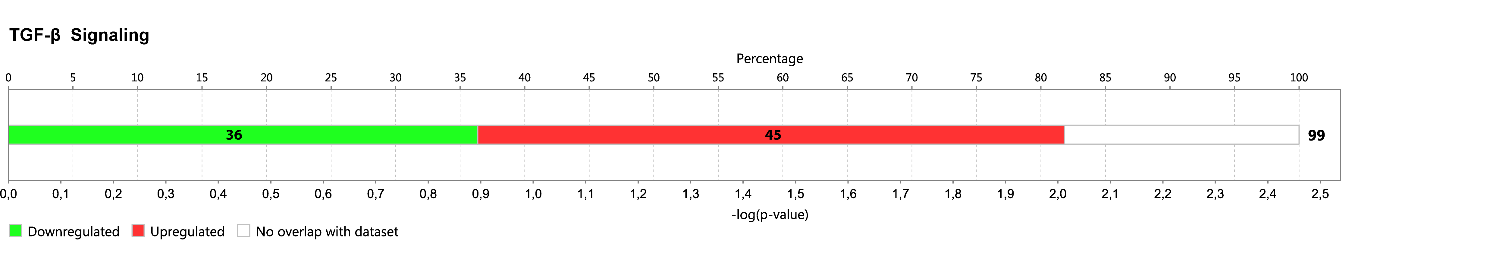
 **Supplementary Figure 5: TGF-β signaling pathway.** TGF-β signaling pathway enrichment at 72h, identified with IPA analysis (72h dyn vs. 72h stat). The number of up-regulated and down-regulated genes belonging to the pathway is indicated respectively by the red and the green bar. The lower axis indicates the adjusted p-value of the enrichment analysis.

Supplementary Tables

**Supplementary Table 1: Patient characteristics**

| Variable | Saphenectomy patients (n=12)  (Mean ± SD) |
| --- | --- |
| Age (y) | 56 ± 16.8 |
| Male, n (%) | 6 (50%) |
| Hypertension, n (%) | 5 (41.7%) |
| Dyslipidaemia, n (%) | 0 (0%) |
| Diabetes, n (%) | 1 (8.3%) |
| Body mass index | 27.6 ± 4.7 |
| Smoking, n (%) | 4 (33.3%) |
| Glycaemia | 110 ± 47 |
| Creatinine | 0.78 ± 0.2 |

**Supplementary Table 2: RNA-Seq outcomes**
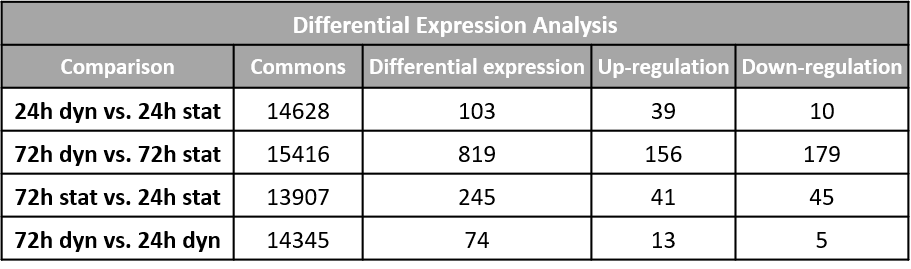

Supplement: Supplementary file 1 [file Data_Sheet_1.docx]
